# Enhanced decision-making through multimodal training

**DOI:** 10.1038/s41539-019-0049-x

**Published:** 2019-08-05

**Authors:** Christopher E. Zwilling, Ana M. Daugherty, Charles H. Hillman, Arthur F. Kramer, Neal J. Cohen, Aron K. Barbey

**Affiliations:** 1 Decision Neuroscience Laboratory, University of Illinois, Urbana, IL USA; 2 Beckman Institute for Advanced Science and Technology, University of Illinois, Urbana, IL USA; 3 Department of Psychology, Wayne State University, Detroit, MI USA; 4 Department of Psychology, Northeastern University, Boston, MA USA; 5 Center for Brain Plasticity, University of Illinois, Urbana, IL USA; 6 Department of Psychology, University of Illinois, Urbana, IL USA; 7 Neuroscience Program, University of Illinois, Urbana, IL USA; 8 Department of Bioengineering, University of Illinois, Urbana, IL USA

**Keywords:** Human behaviour, Decision

## Abstract

A central aim of research in the psychological and decision sciences is to establish interventions that enhance performance, investigating the efficacy of modern approaches to improve human inference and decision-making. Whereas the decision sciences have established interventions to reduce decision biases by promoting strategies for critical thought and reasoning, methods from psychology have instead focused on enhancing cognition through skill-based training of executive functions. Contemporary research in psychology has engaged these operations through multi-modal interventions designed to enhance cognition and physical health through training of executive functions, mindfulness meditation, and physical fitness. Despite the comparable aims of research in the psychological and decision sciences, the efficacy of multi-modal interventions to enhance decision-making remain to be established. We therefore conducted a comprehensive, 16-week, randomized controlled trial (RCT) to investigate this issue, enrolling 160 healthy adults in one of four interventions: (1) high-intensity cardioresistance fitness training (HICRT); (2) HICRT and cognitive training of core executive functions; (3) HICRT and cognitive training, along with mindfulness meditation training; or (4) active control training. The results of our RCT demonstrate that HICRT training and multi-modal interventions that also incorporate cognitive training and mindfulness meditation have beneficial effects on decision-making competence. The observed pattern of findings motivate the application of modern interventions from psychology and cognitive neuroscience to enhance human judgment and decision-making in complex, real-world environments.

## Introduction

Contemporary research in the psychological and decision sciences aims to enhance cognition through the design of experimental protocols that adopt complementary approaches to intervention. In the human judgment and decision-making literature, interventions are designed to reduce decision biases by promoting a reliance upon cognitive systems that facilitate critical thought and deliberation.^1,2^ In contrast, research in cognitive psychology has instead focused on enhancing cognition through skill-based training of executive functions.^3,4^ Modern approaches to intervention from cognitive psychology have engaged these operations through the use of multi-modal interventions that are designed to enhance cognition through the training of executive functions,^5–7^ mindfulness meditation,^8^ and physical activity and aerobic fitness.^9–11^ Despite the comparable aims of each research program and the similar cognitive operations they are designed to enhance,^12^ remarkably little research has been conducted to bridge these areas of investigation. The absence of such data represents a substantial gap in the understanding of the cognitive foundations of human decision-making and the capacity of multi-modal interventions to enhance these functions. We therefore conducted a comprehensive, 16-week, randomized controlled trial (RCT) to evaluate the efficacy of contemporary interventions from cognitive psychology to enhance decision-making.

We investigate decision-making through the lens of the Adult Decision-Making Competence test (A-DMC; 13). The A-DMC is a well-validated test of decision-making whose measures demonstrate internal consistency, high test-retest reliability, and are predictive of real-world decision outcomes, including economic, social, and medical decisions.^13–20^ The human judgment and decision-making literature has established three essential competencies of decision-making that are investigated by the A-DMC, including: (i) *value assessment*, the capacity to assess the value of possible actions and their consequences; (ii) *belief assessment*, the capacity to judge the likelihood or subjective degree of belief in the occurrence of an event; and (iii) *information integration*, the ability to combine available information to make an adaptive choice.^13^ Each of these facets of decision-making represent a different capacity to overcome well-established decision biases and to demonstrate competence in decision-making.

Structural equation modeling of the cognitive abilities underlying decision-making competence further demonstrates that individuals who perform well on the A-DMC tests of information integration and belief assessment also score high on tests of executive function.^21^ Indeed, executive functions are engaged in the service of goal-directed decision-making and enable working memory, response inhibition, and cognitive control (i.e., processes that are associated with value assessment, belief assessment and information integration in decision-making^22^). The observed association between performance on information integration and belief assessment and measures of executive functions motivates the hypothesis that the reduction of decision biases—and corresponding improvements in decision-making competence—can be achieved through interventions designed to enhance executive functions. In addition to strengthening existing associations between decision-making and executive functions (i.e., enhancing information integration and belief assessment), it is possible that uni- and multi-modal training may facilitate decision-making competence by engaging facets of executive function that are not typically recruited, for example, in the case of value assessment measures of the A-DMC.

An independent line of research in cognitive psychology has investigated three primary pathways to improve executive functions: skill-based cognitive training, mindfulness meditation, and physical fitness training. A large empirical literature in psychology has examined the efficacy of cognitive training programs, involving the guided practice of specific cognitive tests to enhance executive functions (for meta-analytic reviews, see refs ^3,4,23^). Prior research indicates that training of executive functions can promote specific cognitive skills (e.g., working memory, response inhibition, and cognitive control), while evidence for generalization beyond the trained task remains the focus of ongoing research and debate.^3,4,23,24^ Accumulating evidence indicates that the neural mechanisms underlying improvements in executive functions are due to changes in functional connectivity, such as increased neural synchrony between frontal and parietal regions.^25^ Potential drivers of functional connectivity include stronger synaptic connections,^26^ increased myelination of the connecting axons,^27^ or increased release rate of dopamine.^28^ The central role of executive functions in decision-making^21^ motivates the application of interventions from cognitive psychology—which are designed to target the cognitive and neural mechanisms underlying executive functions—to enhance decision-making.

An emerging area of research in cognitive psychology investigates the beneficial effects of mindfulness meditation on executive functions. Recent evidence indicates that mindfulness meditation promotes cognitive control^29–31^ and serves to minimize anxiety-related rumination that impairs executive functions.^8,32–35^ Neuroscience evidence further demonstrates that mindfulness meditation induces changes in functional connectivity within networks that support executive functions, including the ventral attention network^36^ (see also refs ^37–39^). These findings suggest that mindfulness meditation enhances the cognitive and neural mechanisms of executive functions and may be further applied to enhance decision-making.

A complementary literature in health psychology investigates the efficacy of moderate intensity physical activity and fitness training to enhance executive functions (for reviews, see refs ^9^^,10^^,40^). A recent meta-analysis demonstrates that physical activity and fitness training confer beneficial effects on executive functions, observing an effect size gain of 0.34 across 36 studies.^40^ A growing body of neuroscience evidence further indicates that fitness training promotes efficient functional connectivity within brain networks for cognitive control, primarily within the fronto-parietal network.^41–43^ For example, a 1-year walking intervention was associated with increased functional connectivity within the fronto-parietal network of healthy older adults and corresponding gains in cognitive control.^44^ Taken together, evidence from health psychology and neuroscience demonstrates that physical fitness training improves executive functions, suggesting that it may also have beneficial effects on decision-making.

The reviewed findings support the efficacy of modern interventions from cognitive psychology to enhance executive functions and motivate their application to the decision sciences. Although uni-modal approaches to intervention are most common, an emerging literature examines the efficacy of multi-modal interventions that are designed to leverage the beneficial effects of multiple intervention modalities. For example, recent evidence demonstrates that multi-modal cognitive and physical fitness training produces greater improvements in executive functions compared to uni-modal training alone.^6,45,46^ Indeed, a recent meta-analysis investigating the combined effects of cognitive and physical fitness training across 20 studies concluded that multi-modal training delivers synergistic effects that enhance performance beyond uni-modal training alone.^47^ While the mechanisms underlying the beneficial effects of multi-modal interventions are still under investigation, animal models suggest that both cognitive and physical fitness training may promote neural plasticity and stimulate neurogenesis.^48^ These findings support the efficacy of multi-modal interventions—providing evidence that a multi-modal approach can enhance performance on tests of executive functions and further motivating their application to the context of decision-making.

The present study therefore sought to investigate the efficacy of modern interventions from cognitive psychology to enhance decision-making, conducting a comprehensive, 16 week RCT (*n* = 160) that administered: (a) high-intensity cardioresistance fitness training (HICRT); (b) HICRT and cognitive training; (c) HICRT and cognitive training, along with mindfulness meditation training; or (d) active control training. We aimed to investigate: (i) whether HICRT can enhance specific facets of decision-making; (ii) whether the combination of cognitive and HICRT is more effective than HICRT alone; and (iii) whether the addition of mindfulness meditation further benefits facets of decision-making.

## Results

### Training efficacy

To evaluate the efficacy of each intervention modality, paired t-tests were used to compare pre- vs. post-intervention training indicators: fat-free VO_2_ max (i.e., the maximum volume of oxygen consumed in milliliters of oxygen per kilogram of lean body mass per minute) for fitness, initial vs. final level of difficulty for cognitive and active control training tasks, and the Mindful Attention Awareness Scale, or MAAS^49^ questionnaire assessing awareness of the present moment for meditation. The pre- and post-intervention means for each indicator are reported in Table 1.

**Table 1:** Pre- and post-intervention training indicators

Indicator	F	F	F + C	F + C	F + C + M	F + C + M	AC	AC
	Pre	Post	Pre	Post	Pre	Post	Pre	Post
Fat-free VO_2_ max	56.8	60.5	57.1	60.6	56.4	59.2	56.9	56.1
Standard deviation	9.7	10.1	7.5	7.5	6.6	6.8	6.8	7.5
Ante up			6	16	6	15		
Standard deviation			1	2	1	2		
Figure Weights			2	16	2	13		
Standard deviation			1	4	1	5		
Dual n-back			2	19	2	15		
Standard deviation			1	8	1	6		
Pen ‘Em Up			2	19	2	16		
Standard deviation			1	6	1	6		
Riding Shotgun			6	10	6	10		
Standard deviation			1	2	1	3		
Pipe Mania			2	12	2	8		
Standard deviation			1	4	1	3		
Supply run			4	17	4	14		
Standard deviation			1	4	1	3		

MAAS					4.26	3.92		
Standard deviation					0.90	0.94		
Change detection 1							3	30
Standard deviation							1	9
Change detection 2							3	42
Standard deviation							1	9
Change detection 3							4	39
Standard deviation							2	9
Visual search 1							4	9
Standard deviation							1	2
Visual search 2							4	21
Standard deviation							1	4
Visual search 3							3	11
Standard deviation							1	3

Mean values, and standard deviation, for training indicators for each group at pre- and post-intervention. ‘F’ denotes the fitness training intervention; ‘F + C’ denotes the fitness plus cognitive training intervention; ‘F + C + M’ denotes the fitness plus cognitive training plus meditation intervention; and ‘AC’ denotes the Active Control. Fat-free VO_2_ max is the maximum volume of oxygen consumed in milliliters of oxygen per kilogram of lean body mass per minute. MAAS stands for Mindful Attention Awareness Scale. For the cognitive and active control training, the means and standard deviations are rounded to the nearest integer. These integer values represent levels of training difficulty, with 1 representing the easiest level, which was also the starting level for all participants. For the cognitive and active control training, the value listed at Pre represents the mean level of difficulty across all participants for the first session while the value listed at Post represents the mean level of difficulty for the final session. Also see Supplementary Table 3 for more details about the *Mind Frontiers* training tasks

Each intervention that engaged in HICRT demonstrated improvements in cardiorespiratory fitness, as measured by fat-free VO_2_ max (i.e., the maximum volume of oxygen consumed in milliliters of oxygen per kilogram of body weight per minute) (F: *t* = 4.02, *p* = 0.0003, *df* = 32; F + C: *t* = 4.24, *p* = 0.0001, *df* = 40; F + C + M: *t* = 3.36, *p* = 0.002, *df* = 38; see Table 1). Importantly, the active control did not demonstrate an improvement in fat-free VO_2_ max (*t* = 1.27, *p* = 0.21, *df* = 38). Both intervention groups that completed cognitive training (i.e., F + C and F + C + M) improved for all seven cognitive training tasks (all *p* < 0.0001). The learning curves are presented in Supplementary Fig. 4. The active control group, which received training in three change detection tasks and three visual search tasks, also improved on all six training tasks (*p* < 0.0001). The learning curves are presented in Supplementary Fig. 5. Finally, the efficacy of the mindfulness meditation intervention was measured by the Mindful Attention Awareness Scale, or MAAS. MAAS assesses awareness of and attention to the present moment, skills that reliably improved in the intervention group that completed mindfulness meditation (*t* = 3.56, *p* = 0.001, *df* = 38).

### Executive functions

Having established the efficacy of training for each intervention, we next examine whether the observed training improvements are associated with enhanced executive functions (i.e., transfer of training to executive functions). We applied a well-established executive function measure that examines *mental set shifting*, the capacity to adaptively shift attention from one stimulus attribute to another.^50^ Mental set shifting is a hallmark of goal-directed behavior and supports the updating of cognitive control parameters to accommodate changing task demands. This capacity was indexed by the switch cost measure of the set shifting task, which examines the participant’s ability to shift their attention to a different attribute in a visual array of stimuli, such as the color, size, or number of shapes^50^ (see Supplementary Fig. 1). There were no differences between groups at pre-intervention in the switch cost measure (*F* = 0.75, *p* = 0.52, *df* = (3,156)). The ANCOVA model testing the post-intervention switch cost measure of executive function, while controlling for the pre-intervention switch cost measure, was significant (*F* = 4.17, *p* = 0.0072, *df* = (4,155), Table 2). The uni- and multi-modal interventions demonstrated gains in the switch cost measure of executive function at post-intervention, relative to pre-intervention, whereas the active control demonstrated a decline in the switch cost measure. These results provide evidence that the uni- and multi-modal training interventions led to improvements in executive function.

**Table 2:** Descriptive and inferential statistics for the switch cost measure of executive function

Statistic	F	F + C	F + C + M	AC
Pre-intervention mean (SD)	78.97 (3.1)	78.17 (3.4)	78.83 (3.28)	78.07 (3.4)
Post-intervention mean (SD)	80.31 (3.8)	79.02 (2.8)	79.10 (3.94)	77.42 (3.8)
Beta coefficients	2.89	1.60	1.68	–
*p*-value	0.00064*	0.043*	0.038*	–
Cohen’s d effect size	0.68	0.29	0.31	–

‘F’ denotes the fitness training intervention; ‘F + C’ denotes the fitness plus cognitive training intervention; ‘F + C + M’ denotes the fitness plus cognitive training plus meditation intervention; and ‘AC’ denotes the active control. AC was the reference group in the ANCOVA model so the beta coefficients, *p*-values and effect sizes represent changes, relative to the active control. *p*-values with an * are significant at the 0.05 threshold

### Processing efficiency

To further investigate the specificity of transfer, we examined performance on cognitive tests that were predicted to improve only in the active control group. The active control intervention was designed to increase the speed at which participants correctly solve visual search and change detection tasks and, consequently, to enhance performance on tests of processing efficiency.^51^ To investigate this hypothesis, we administered three tests of processing efficiency: *Pattern Comparison*,^51^ *Letter Comparison*,^51^ and the *Digit Symbol Substitution Task*^52^ (also see Supplementary Fig. 2). There were no differences between groups at pre-intervention in the number of items correct for all measures of processing efficiency (*Pattern Comparison: F* = 0.09, *p* = 0.97, *df* = (3,148); *Letter Comparison: F* = 0.31, *p* = 0.82, *df* = (3,139); and *Digit Symbol Substitution Task F* = 0.44, *p* = 0.73, *df* = (3,149); see Table 3) and no group differences at pre-intervention for the two measures of processing efficiency for which reaction time was recorded (*Pattern Comparison: F* = 0.39, *p* = 0.76, *df* = (3,148) and *Letter Comparison: F* = 0.15, *p* = 0.93, *df* = (3,139); see Table 3). The only within group differences, comparing pre-intervention to post-intervention reaction time, were observed for the active control for the two processing efficiency tests that included reaction time measures (*Pattern Comparison: t* = 2.40, *p* = 0.02, *df* = 39 and *Letter Comparison: t* = 2.20, *p* = 0.03, *df* = 37; see Table 3). Only the active control had marginal within group differences, comparing the number of items correct at pre-intervention to post-intervention, for all three measures of processing efficiency (*Pattern Comparison: t* = 1.82, *p* = 0.07, *df* = 39; *Letter Comparison: t* = 1.62, *p* = 0.11, *df* = 37; and *Digit Symbol Substitution Task t* = 1.69, *p* = 0.09, *df* = 40; see Table 3). Active control training was associated with a reliable increase in the number of items correct from pre- to post-intervention for *Letter Comparison* (*F* = 3.41, *p* = 0.020, *df* = (4,138)) and a reliable decrease in reaction time from pre- to post-intervention for *Pattern Comparison* (*F* = 2.89, *p* = 0.037) and *Letter Comparison* (*F* = 4.06, *p* = 0.008, *df* = (4,138); see Table 3). These results for the active control are relative to the uni- and multi-modal intervention groups in an ANCOVA model, controlling for performance at pre-intervention. Applying the same model, there was not a reliable increase in the number of items correct from pre- to post-intervention for *Pattern Comparison* (*F* = 1.15, *p* = 0.33, *df* = (4,147)) and the *Digit Symbol Substitution Task* (*F* = 0.78, *p* = 0.51, *df* = (4,148)) for the active control. Taken together, these findings provide evidence that the active control training improved processing efficiency.

**Table 3:** Number of items correct and reaction time statistics for processing efficiency

Statistics for each measure	F	F + C	F + C + M	AC
Pattern Comparison				
Items correct				
Pre-intervention mean (SD)	40.6 (7.8)	41.6 (8.3)	41.1 (7.1)	41.1 (9.0)
Post-intervention mean (SD)	42.2 (7.9)	43.2 (7.5)	41.8 (8.7)	44.4 (7.0)
Beta coefficients	−1.93	−1.45	−2.62	–
*p*-value	0.20	0.32	0.076	–
Cohen’s d effect size	0.13	0.07	0.24	–
Reaction time				
Pre-intervention mean (SD)	1292 (369)	1271 (313)	1236 (243)	1311 (339)
Post-intervention mean (SD)	1244 (312)	1213 (297)	1278 (361)	1154 (236)
Beta coefficients	99.7	80.7	165	–
*p*-value	0.09	0.15	0.0041*	–
Cohen’s d effect size	0.23	0.16	0.53	–

Letter Comparison				
Items correct				
Pre-intervention mean (SD)	24.9 (3.9)	25.3 (5.5)	25.9 (4.7)	25.6 (4.5)
Post-intervention mean (SD)	24.8 (4.5)	25.9 (4.6)	25.4 (3.7)	27.3 (4.4)
Beta coefficients	−2.02	−1.20	−2.12	–
*p*-value	0.0098*	0.11	0.0052*	–
Cohen’s d effect size	0.49	0.21	0.53	–
Reaction time				
Pre-intervention mean (SD)	2099 (396)	2116 (479)	2048 (531)	2095 (365)
Post-intervention mean (SD)	2140 (469)	2056 (408)	2069 (412)	1918 (321)
Beta coefficients	219	124	180	–
*p*-value	0.0016*	0.059	0.0072*	–
Cohen’s d effect size	0.65	0.28	0.50	–
Pre-intervention mean (SD)	40.6 (9.1)	40.3 (7.8)	41.1 (7.1)	40.8 (9.1)

DSST				
Items correct				
Pre-intervention mean (SD)	94.2 (11.0)	94.2 (13.2)	95.2 (12.1)	92.1 (12.1)
Post-intervention mean (SD)	98.3 (12.6)	96.9 (12.8)	100.5 (13.0)	96.9 (12.5)
Beta coefficients	−0.29	−1.68	1.09	–
*p*-value	0.88	0.35	0.56	–
Cohen’s d effect size	0.00	0.06	0.02	–

‘F’ denotes the fitness training intervention; ‘F + C’ denotes the fitness plus cognitive training intervention; ‘F + C + M’ denotes the fitness plus cognitive training plus meditation intervention; and ‘AC’ denotes the active control. AC was the reference group in the ANCOVA model so beta coefficients, *p*-values and effect sizes represent changes, relative to the active control. Results with an * denote a significant *p*-value at the 0.05 threshold. DSST is Digit Symbol Substitution Task. Reaction time is measured in milliseconds. Reaction time was not measured for DSST

We performed an analysis to examine the role of speed-accuracy tradeoffs for each intervention group. Specifically, we combined the mean number of items correct and reaction times for *Pattern Comparison* and *Letter Comparison* from Table 3. On average, the fitness group answered 1.5 more correct responses at post-intervention than pre-intervention and were 7 ms faster for each response at post-intervention. The fitness plus cognitive training group answered 2.2 more correct responses at post-intervention than pre-intervention and were 118 milliseconds faster for each response at post-intervention. The fitness plus cognitive training plus meditation group answered 0.2 more correct responses at post-intervention than pre-intervention and took 63 milliseconds longer for each response at post-intervention. The active control group answered 5 more correct responses at post-intervention than pre-intervention and took 334 milliseconds less for each response at post-intervention. Thus, the observed pattern of results does not support a speed-accuracy tradeoff hypothesis (i.e., improvements in speed do not come at the expense of accuracy).

Jointly considering the processing efficiency and executive function results, we draw the following conclusions. First, active control training produced improvements in processing efficiency, but not executive function. Second, the uni- and multi-modal interventions improve executive function, but not processing efficiency. Finally, this pattern of results provides evidence for selectivity of training in the active control group and in the uni- and multi-modal interventions.

### Decision-making competence

Having established the specificity of uni- and multi-modal interventions for improved executive functions, we next examined the hypothesis that these interventions also enhance decision-making competence. Three facets of decision-making competence were investigated: value assessment, belief assessment and information integration (see Supplementary Note 2). No differences among the intervention groups were observed at pre-intervention (value assessment: *F* = 0.81, *p* = 0.49*, df* = (3,133); belief assessment: *F* = 0.31, *p* = 0.82*, df* = (3,133); Information Integration: *F* = 0.32, *p* = 0.81*, df* = (3,133)). At the omnibus level, all three facets of decision-making competence demonstrated improvements from pre- to post-intervention (value assessment: *F* = 28.1, *p* < 0.0001*, df* = (4,132); belief assessment: *F* = 8.41, *p*<0.0001*, df* = (4,132); information integration: *F* = 8.8, *p* < 0.0001*, df* = (4,132); see Table 4). Using a Bonferroni corrected threshold (i.e., 0.05/3 = 0.017), all results remain significant. The uni-modal HICRT intervention improved belief assessment (*p* = 0.0007, Cohen’s *d* = 0.73*, df* = (4,132)) and Information Integration (*p* = 0.012, Cohen’s *d* = 0.47*, df* = (4,132)). The multi-modal HICRT plus cognitive training intervention enhanced value assessment (*p* = 0.025, Cohen’s *d* = 0.38*, df* = (4,132)) and belief assessment (*p* = 0.022, Cohen’s *d* = 0.40*, df* = (4,132)). The multi-modal HICRT plus cognitive training plus meditation intervention improved value assessment (*p* = 0.0018, Cohen’s *d* = 0.62*, df* = (4,132)). The results of all other analyses examining intervention effects were null (*p* *>* 0.05). These include HICRT and value assessment, HICRT plus cognition and information integration, and HICRT plus cognitive training plus meditation and belief assessment and Information Integration. Because the active control group demonstrated declines from pre- to post-intervention in all three measures of decision-making competence, the analyses presented in Table 4 were repeated without the active control group, instead using HICRT as the comparison group. This represents an experimentally rigorous comparison given that all three interventions administered HICRT, enabling an assessment of the selective effects of each intervention modality that accompanied physical fitness training. The results of this analysis are presented in Supplementary Table 4 and are consistent with the findings in Table 4, suggesting that the decrease in decision-making competence in the active control group does not drive the improvements in value assessment, belief assessment, and information integration reported here.

**Table 4:** Descriptive and inferential statistics for three facets of decision-making competence

Statistic	F	F + C	F + C + M	AC
Value assessment				
Pre-intervention mean (SD)	4.25 (0.38)	4.31 (0.42)	4.21 (0.33)	4.35 (0.49)
Post-intervention mean (SD)	4.19 (0.51)	4.38 (0.46)	4.38 (0.32)	4.23 (0.42)
Beta coefficients	0.026	0.17	0.24	–
*p*-value	0.75	0.025*	0.0018*	–
Cohen’s d effect size	0.00	0.38	0.62	–
Belief assessment				
Pre-intervention mean (SD)	0.73 (0.085)	0.72 (0.077)	0.71 (0.092)	0.73 (0.079)
Post-intervention mean (SD)	0.76 (0.061)	0.74 (0.082)	0.72 (0.062)	0.70 (0.085)
Beta coefficients	0.06	0.04	0.02	–
*p*-value	0.0007*	0.022*	0.17	–
Cohen’s d effect size	0.73	0.40	0.16	–
Integration				
Pre-intervention mean (SD)	0.87 (0.103)	0.88 (0.095)	0.87 (0.108)	0.87 (0.103)
Post-intervention mean (SD)	0.91 (0.085)	0.89 (0.072)	0.87 (0.099)	0.85 (0.104)
Beta coefficients	0.05	0.03	0.02	–
*p*-value	0.012*	0.14	0.37	–
Cohen’s d effect size	0.47	0.18	0.06	–

‘F’ denotes the fitness training intervention; ‘F + C’ denotes the fitness plus cognitive training intervention; ‘F+C+M’ denotes the fitness plus cognitive training plus meditation intervention; and ‘AC’ denotes the active control. AC is the reference group in the ANCOVA model so all model betas, *p*-values and effect sizes represent changes, relative to the fitness group. Results with an * denote a significant *p*-value at the 0.05 threshold

### Executive function and decision-making competence

To further elucidate the relationship between executive function and facets of decision-making competence, change scores were computed for the uni- and multi-modal intervention groups. A change score, also known as a delta score, is the post-intervention mean minus the pre-intervention mean. Delta scores were computed from the switch measure of executive function in Table 2 and are presented in Table 5. Delta scores were also computed for each of the three facets of decision-making competence and are presented in Table 5. From the delta scores the slope was computed, which is a ratio of the respective changes in facets of decision-making competence and executive function. This slope represents the rate of change between decision-making competence and executive function and are presented in Table 5. A slope with a larger magnitude represents a stronger relationship between the change in decision-making competence and executive function.

**Table 5:** Changes in decision-making competence and executive functioning

Statistic	F	F + C	F + C + M	AC
Value assessment (VA)				
Delta VA	−0.06	0.07	0.17	−0.11
Delta EF	1.34	0.86	0.28	−0.65
Slope	−0.04	0.08	0.63	0.17
Belief assessment (BA)				
Delta BA	0.04	0.02	0.01	−0.02
Delta EF	1.34	0.86	0.28	−0.65
Slope	0.03	0.03	0.03	0.03
Integration (I)				
Delta I	0.02	0.00	0.00	−0.02
Delta EF	1.34	0.86	0.28	−0.65
Slope	0.02	0.00	0.00	0.03

‘F’ denotes the fitness training intervention; ‘F + C’ denotes the fitness plus cognitive training intervention; ‘F + C + M’ denotes the fitness plus cognitive training plus meditation intervention; and ‘AC’ denotes the active control. Delta represents a change score obtained by subtracting the pre-intervention mean from the post-intervention mean

*VA* value assessment, *BA* belief assessment, *I* integration, *EF* executive function

The results in Table 5 are consistent with the patterns of significance presented in Table 4 but go further by demonstrating how these improvements in decision-making relate to executive function. For instance, the largest slope for value assessment, 0.63, is for the fitness plus cognitive training plus meditation group (F + C + M; see Table 5). This same intervention group demonstrates the largest improvement in value assessment, with an effect size of 0.62 (see Table 4). Fitness plus cognitive training demonstrates the second largest slope (0.08; Table 5) and the second largest effect size (0.38; Table 4). And the fitness only intervention has a negative slope (Table 5) and an effect size of 0.00 (Table 4).

**Fig. 1:**
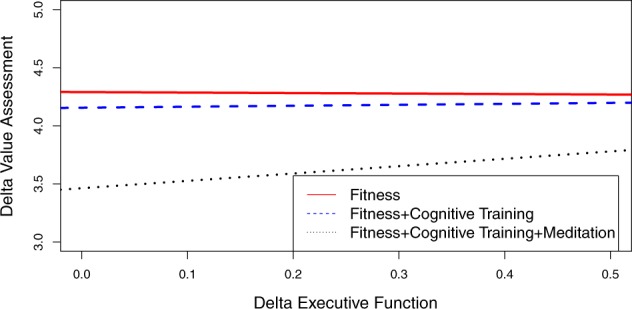
Plot of slopes for value assessment and executive function for the uni- and multi-modal interventions. The positive slope of 0.63 for the fitness plus cognitive training plus meditation intervention condition, indicates improvements in executive function (*x*-axis) are related to improvements in value assessment (*y*-axis). The fitness and fitness plus cognitive training intervention conditions are represented by lines with flat slopes, indicating there were not improvements in executive function related to improvements in value assessment

**Fig. 2:**
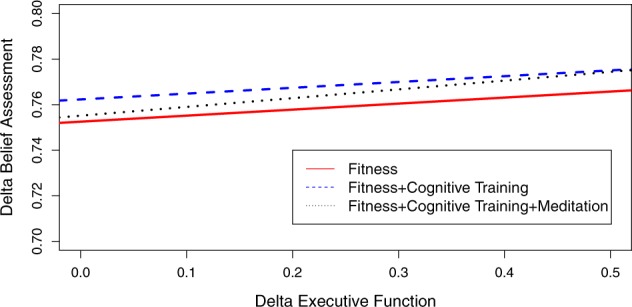
Plot of slopes for belief assessment and executive function for the uni- and multi-modal interventions. All three intervention conditions have positive slopes, indicating some improvements in executive function (*x*-axis) related to improvements in value assessment (*y*-axis). The fitness group has the steepest slope and largest intercept, indicating greater improvements in executive function related to improved value assessment. The fitness plus cognitive training intervention had the 2nd largest intercept and the fitness plus cognitive training plus meditation intervention condition had the smallest intercept

**Fig. 3:**
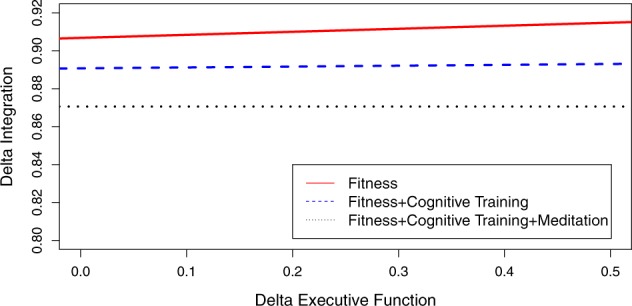
Plot of slopes for integration and executive function for the uni- and multi-modal interventions. Only the fitness condition has a positive slope, indicating improvements in executive function (*x*-axis) relate to improvements in integration (*y*-axis). The fitness plus cognitive training intervention has a slope of 0 and the 2nd largest intercept. The fitness plus cognitive training plus meditation intervention condition also has a slope of 0 and the smallest intercept, indicating there is a weak to non-existent relationship between integration and executive function

Figures 1, 2 and 3 plot the slopes for each of the uni- and multi-modal intervention conditions for value assessment (Fig. 1), belief assessment (Fig. 2) and information integration (Fig. 3). The slope with the largest magnitude in Table 5 for value assessment (0.63) is the HICRT plus cognitive training plus meditation intervention. This slope is plotted in Fig. 1 and demonstrates that the change in executive function was strongest for this intervention group, which also showed the largest effect size gain for the value assessment decision-making measure in Table 4. The HICRT intervention was not related to improvements in value assessment (Table 4) and, as its negative slope (−0.04) in Fig. 1 indicates, there were no accompanying executive function improvements associated with this intervention. The HICRT plus cognitive training intervention demonstrated the second largest gain in value assessment and has the second largest slope (0.08) in Fig. 1, indicating that the more moderate gains in value assessment were related to more moderate gains in executive function.

Figure 2 plots the uni- and multi-modal interventions as a function of changes in belief assessment and executive function. All slopes in Fig. 2 are similar and only differ with respect to their intercepts. The HICRT condiction has the largest intercept and also demonstrates the largest improvement in belief assessment in Table 4, with an effect size of 0.73. The HICRT plus cognitive training plus meditation intervention, which has the weakest effect for belief assessment in Table 4 (0.16), has the second largest intercept. And the HICRT plus cognitive training has the smallest intercept. In Fig. 3, the HICRT intervention has the largest slope and intercept and the largest effect size for information integration in Table 4 (0.47). The HICRT plus cognitive training intervention has the second largest information integration effect size in Table 4 (0.18) and a slope and intercept that is smaller than the HICRT intervention. The HICRT plus cognitive training plus meditation intervention has the smallest slope and intercept in Fig. 3 and the smallest information integration effect size in Table 4.

In sum, the rate of change between facets of decision-making competence and executive function demonstrates that improvements in executive function may underlie the observed enhancements in decision-making. We also examined correlations between these cognitive processes at pre-intervention and observed associations only for information integration and the set shifting measure of executive function in the full sample (value assessment: *r* = 0.03, *p* = 0.73, *n* = 137; belief assessment: *r* = 0.16, *p* = 0.06, *n* = 137; information integration: *r* = 0.24, *p* = 0.004, *n* = 137).

### Expectancies

We investigated the participant’s beliefs about the effectiveness of each intervention at pre-intervention, in an effort to examine whether their expectancies differed for each intervention modality and therefore systematically biased the results.^53^ For instance, a participant randomly assigned to the cognitive training intervention may believe that cognitive training can enhance mental ability and this expectancy alone may lead to cognitive improvements, independent of the intervention protocol. As Table 6 illustrates, expectancies did not differ by intervention group, suggesting that prior beliefs about intervention efficacy did not bias the findings.^24^

**Table 6:** Expectancy analysis

Intervention	Cognitive Training	Exercise	Meditation
F	1.62	1.54	1.70
F + C	1.49	1.36	1.57
F + C + M	1.40	1.46	1.67
Active control	1.57	1.47	1.60
*F* statistic	1.25	0.78	0.52
*p-*value	0.29	0.50	0.67

‘F’ denotes the fitness training intervention; ‘F + C’ denotes the fitness plus cognitive training intervention; and ‘F + C + M’ denotes the fitness plus cognitive training plus meditation intervention. Values in each of three columns of the table are the mean score to responses for one of 3 questions asked at pre-intervention: Does video game training improve cognitive ability? Does engaging in exercise improve cognitive ability? Does practicing meditation improve cognitive ability? To each of these 3 questions, a respondent could ‘Agree’ (Likert score of 1), ‘Neither agree or disagree’ (Likert score of 2) or ‘Disagree’ (Likert score of 3). The F statistic in each column is a one-way ANOVA, with intervention as the group factor, indicating whether there are intervention group differences in expectancies. No results are statistically significant

## Discussion

We conducted a RCT in healthy adults (*n* = 160) to investigate the hypothesis that modern interventions from cognitive psychology known to strengthen executive functions would enhance specific facets of decision-making competence. Improvements in executive functions were predicted to enhance decision-making by promoting critical thought and deliberation (i.e., cognitive control), and enabling the respondent to overcome well-established biases in decision-making.^4,40,54^ The present study represents one of the largest and most comprehensive multi-modal intervention trials conducted to date and supports three primary conclusions.

First, we observed significant improvements in decision-making competence within each of the uni- and multi-modal interventions (controlling for performance at pre-intervention and with respect to the active control group). Notably, the pattern of improvements were selective, demonstrating enhanced executive functions (as assessed by the switch cost measure of the SST) and improvements in specific facets of decision-making competence (as measured by the A-DMC). This finding reflects the importance of mental operations for cognitive control—captured in cognitive psychology by measures of executive functions (i.e., mental set shifting) and in the decision sciences by tests of decision-making competence (i.e., value assessment, belief assessment, and information integration). This pattern of findings motivates a multidisciplinary approach to improve decision-making, demonstrating that contemporary interventions from cognitive psychology can be productively applied to enhance executive functions and promote competence in decision-making.

Second, our study provides evidence that HICRT is a primary driver of improvements in decision-making competence. Indeed, among the administered interventions, HICRT demonstrated the largest beneficial effects on belief assessment (*d* *=* 0.73), information integration (*d* *=* 0.47), and Switch Cost (*d* *=* 0.68). As Figs 2 and 3 illustrate, the largest improvements in executive function are related to these beneficial effects on decision-making. Moreover, the observed improvements in decision-making competence for the uni-modal HICRT intervention exhibit a dose-response effect. Specifically, belief assessment demonstrates a 0.73 effect size gain for the fitness intervention with 48 sessions of HICRT and a 0.40 effect size gain for the fitness plus cognitive training intervention with 28 sessions of HICRT. These findings extend prior research supporting the beneficial effects of HICRT on executive function,^40^ setting the stage for future physical fitness interventions that aim to enhance decision-making skills that selectively decline in cognitive aging and are symptomatic in psychiatric illness and neurological disease.

Third, we found that multi-modal interventions have beneficial effects on decision-making competence beyond those conferred by HICRT alone. Multi-modal HICRT plus cognitive training, and multi-modal HICRT plus cognitive training plus meditation improved value assessment (*d* = 0.38 and *d* = 0.62; respectively). The direct comparison of these multi-modal interventions permit an investigation of the unique contribution of mindfulness meditation, demonstrating that meditation conferred an additional benefit to value assessment (i.e., with an effect size improvement of 0.62 vs. 0.38). Accumulating evidence indicates that mindfulness meditation promotes cognitive control^29^ and serves to minimize anxiety-related rumination that impairs executive functions.^8,32–35^ Recent work demonstrates that mindfulness meditation reduces decision bias,^55^ providing direct evidence to support the beneficial effects of mindfulness meditation on decision-making (see Supplementary Table 2).

Although the current study represents one of the largest and most comprehensive multi-modal trials conducted to date, it is important to present our findings in the light of several limitations. First, the observed level of attrition was higher than studies that have only a single session or that administer a single intervention modality and reflects the significant time commitment (i.e., 60 h) and task demands required to complete the study (i.e., receiving training for up to three intervention modalities). We conducted further analyses to investigate whether attrition introduced systematic bias in the study results (see Supplementary Note 4 and Supplementary Fig. 3). The results of this analysis demonstrate that attrition did not introduce systematic bias in the study findings (i.e., with respect to participant characteristics and the level of attrition between groups). Nonetheless, future multi-modal intervention trials should employ methods to further reduce the attrition rate and to investigate factors that contribute to an increase in attrition in comparison to standard, uni-modal intervention trials. Second, the experimental design of the present RCT did not enable an investigation of the independent and joint contributions of each intervention modality, constraining the inferences that can be drawn about the mechanisms underlying their effects. Although the current findings support inferences about selective effects of HICRT, the other intervention modalities were not administered independently and therefore an examination of their contributions in isolation is not possible. Additionally, even though the uni- and multi-modal interventions produce significant effect size gains in decision-making in the current study, more intervention studies designed to improve decision-making are needed. Therefore, future multi-modal trials should aim to replicate the current results and further investigate the independent and joint contributions of each intervention modality to further characterize their role in executive function and decision-making. Third, while the declines in decision-making competence within the active control condition do not influence the reported results, future research should carefully examine the implications of active control training within this context. In the present study, active control training facilitated processing efficiency and, as a consequence, may have promoted fast, automatic responses that are known to reduce decision-making competence (i.e., engaging intuitions rather than slow, deliberate processes for critical thought and evaluation). Fourth, the current study investigated decision-making improvements with one executive function test: mental set-shifting. Future research should consider multiple tests of executive function to more comprehensively examine whether the effects of training are specific to this measure or benefit executive functions more broadly.^56^ Additionally, while set shifting represents a canonical measure of cognitive flexibility, executive function represents a wide range of cognitive processes (e.g., inhibition and working memory^22^). Future research should therefore also investigate whether the observed improvements in decision-making competence reflect specific facets of executive function. Fifth, the present findings motivate the development of a more precise mechanistic model of the cognitive and neurobiological processes that are enhanced by uni- and multi-modal training, and the further characterization of their roles in executive functions and decision-making. The current findings suggest that modern interventions from cognitive psychology can be productively applied to enhance decision-making competence and provide evidence that their beneficial effects derive from the engagement of mechanisms for executive functions (i.e., cognitive control). Thus, the present findings set the stage for multidisciplinary research in the psychological and decision sciences that aims to further measure, model, and characterize the beneficial effects of uni- and multi-modal interventions on decision-making.

In conclusion, research in the psychological and decision sciences aims to enhance human judgment and decision-making. Historically, however, research in these fields has largely progressed along independent lines of investigation. The present study was motivated by an effort to integrate research across disciplines, conducting a comprehensive, 16-week, randomized controlled trial (*n* = 160) to evaluate the efficacy of modern interventions from psychology to enhance decision-making. The results demonstrate that multiple intervention modalities have beneficial effects on decision-making competence. We observed that uni-modal fitness training produced significant effect size improvements in belief assessment and information integration. Our study further demonstrated that multi-modal interventions that include fitness training, cognitive training, and mindfulness meditation significantly improved value assessment. Finally, multi-modal fitness plus cognitive training improved value and belief assessment. Thus, our findings support the efficacy of a multidisciplinary approach and motivate the application of modern interventions from psychology and cognitive neuroscience to enhance judgment and decision-making in complex, real-world environments.

## Methods

### Experimental design

The experimental protocol was approved by the University of Illinois Institutional Review Board (IRB). Study participants were recruited from the Urbana-Champaign, Illinois community and provided informed written consent in accordance with the University of Illinois IRB. Demographics of the 160 study participants are presented in Table 7.

**Table 7:** Study demographic

Demographic	Value
Age	
Range	18–42
Average	23.8
Sex	
Male	48%
Female	52%
Highest education level	
High school diploma	5.2%
Enrolled in college	48.4%
Completed college	16.1%
Graduate or professional	30.3%

Study eligibility required participants to: (a) be 18–44 years of age; (b) have at least a high school diploma; (c) speak English fluently; (d) have normal or corrected-to-normal vision and hearing; (e) not have current or recent medications affecting the central nervous system; (f) not have a history of psychological, neurological, or endocrine disease; (g) not have had a concussion within the past 2 years; (h) not have learning disorders; (i) to not smoke more than 10 cigarettes per day; (j) to have a body mass index under 35; and (k) to have at least one positive response on the revised Physical Activity Readiness Questionnaire.^57^

Participants were randomly assigned to one of four intervention groups: (a) HICRT; (b) multi-modal HICRT plus cognitive training; (c) multi-modal HICRT, cognitive, and mindfulness meditation training; and (d) active control training. The activities within an intervention protocol followed the same order for each training session. The training portion of the study lasted 16 weeks and each week comprised three, 70-min, intervention sessions for a total of 48 sessions. The multi-modal interventions combined the training protocols in a specific manner, beginning first with an intervention modality that was designed to prime the brain for new skill learning. The order and frequency of administration of each training activity is listed in Supplementary Table 1. For the multi-modal interventions, participants trained on one type of activity (i.e., fitness, cognitive training or meditation) for a given session. The specific training activities included in each intervention group are now reviewed. Please see Supplementary Note 1 for further detail of all training activities.

### High-intensity cardioresistance fitness training (HICRT)

The HICRT intervention was supervised by professional fitness trainers, who led each 70-min fitness session.^11^

### Cognitive training

Participants in the cognitive training session used a tablet computer to play *Mind Frontiers*,^6,7^ a suite of seven Western-themed cognitive tasks with adaptive difficulty. Table 8 lists each task along with cognitive abilities targeted, including executive functions, visuospatial working memory, and analogical reasoning. Further description of the cognitive training intervention is provided in Supplementary Table 3.

**Table 8:** Summary of *Mind Frontiers* multi-task cognitive training platform

Mind Frontiers	Cognitive domain	References
Ante Up	Executive function	^58, 59^
The Irrigator	Visuospatial reasoning	^60^
Pen ‘Em Up	Executive function; dual task switching	^61^
Riding Shotgun	Visuospatial working memory	^62, 63^
Sentry Duty	Working memory	^64, 65^
Supply Run	Executive function	^66^
Trader Jack’s	Analogical reasoning	^67^

### Mindfulness meditation training

The mindfulness meditation intervention was supervised by professional meditation and yoga instructors, who led each 70-minute mindfulness meditation session.

### Active control training

Participants in the active control group engaged in computerized training tasks based on visual search and change detection training for 48 sessions over 16 weeks.^65,68^ The active control tasks were administered via tablet computers. The change detection task required the participant to identify the item that changed between two arrays of objects (i.e., cars, toys or street signs). Task difficulty increased with decreasing presentation time and/or increasing the number of objects in the array. The visual search task required participants to search for a target (i.e., F, P or a hand) among distractors. Difficulty was raised by increasing the number and/or heterogeneity of the distractors.

The cognitive performance measures administered to assess transfer of training are summarized below.

### Set shifting task (SST)

The switch cost measure of the SST^50^ was used to investigate mental set shifting, which represents a core executive function. Each trial of the SST consisted of a fixation cross in the center of the screen, followed by a cue to pay attention to one attribute—color, shape or size—and, finally, the visual stimuli array. In a non-switch trial, the cued attribute does not change between two trials. In a switch trial, the cued attribute changes between two trials. The switch cost measure of executive functions in the SST was determined by subtracting the number of correct responses on all non-switch trials from the number of correct responses on all switch trials. Please see Supplementary Note 3 and Supplementary Fig. 1 for further detail.

### Processing efficiency

Processing efficiency was assessed by three neuropsychological tests: *Pattern Comparison*, *Letter Comparison*, and the *Digit Symbol Substitution Test*. Please see Supplementary Note 3 and Supplementary Fig. 2 for further detail.

### Adult decision-making competence (A-DMC)

We administered a well-validated battery of measures to investigate three facets of decision-making, employing the Adult Decision-making Competence test.^13–20^ The A-DMC was administered at pre- and post-intervention. The three facets of decision-making competence (value assessment, belief assessment, and information integration) are calculated by taking the average of the performance measure of each A-DMC subtest within that measure. Please see Supplementary Note 2 for further detail.

The statistical software used, models employed, and data analysis techniques are described below.

### Statistical software and packages

All data analyses were conducted using the R Studio interface Version 1.0.143, which runs on top of the base R installation Version 3.4.2.^69,70^ Several packages were used for the analyses, including: *car, reshape, plyr*, and *compute.es*.^71–75^

### Descriptive statistics

All tables of results contain the pre- and post-study means, along with the standard deviations.

### *t*-test and ANOVA

*t*-tests were used examine changes from pre- to post-test, within intervention groups and were always two-sided. One-way ANOVA models were used to test for group differences at pre-intervention.

### Analysis of covariance (ANCOVA)

The ANCOVA was the primary statistical model used to examine pre- to post-intervention improvements. The active control represents the comparison group for each of intervention. The ANCOVA model includes baseline performance as a covariate, while testing for group differences at post-study (with respect to the active control). In R, the ANCOVA model yields an omnibus test statistic for the overall model fit in addition to regression parameter estimates three planned contrasts: fitness vs. active control, fitness plus cognitive training vs. active control and fitness plus cognitive training plus meditation vs. active control.

*Cohen’s d*. Cohen’s d is a commonly used effect size measure and is provided in conjunction with p-values.

### Data quality

Data points exceeding the Tukey criteria of 1.5 times the interquartile range were not analyzed in an effort to ensure data quality (i.e., to satisfy model assumptions and to prevent individual extreme values from skewing group averages).

## Supplementary information

Intervention protocol

## Data Availability

The individual de-identified participant data and related study documents can be made available upon request.

## Supplementary information

**Supplementary information** accompanies the paper on the *npj Science of Learning* website (10.1038/s41539-019-0049-x).

## Code availability

All analyses were conducted in R using standard packages and functions (see “Methods” section).
