# Supplementary material for: Enhanced decision-making through multimodal training
Source: NPJ Sci Learn. 2019 Aug 5;4:11. doi: 10.1038/s41539-019-0049-x (PMC6683193; doi:10.1038/s41539-019-0049-x)
Supplement: Supplementary file 1 — Intervention protocol [file 41539_2019_49_MOESM1_ESM.docx]

Supplementary Note 1.

**Intervention protocol.**

High-intensity cardioresistance fitness training (HICRT)*.* Fitness trainers supervised a group of 5 participants at each aerobic fitness training session. Warm-up comprised the first 10 minutes of the session and entailed light jogging, walking kicks, walking lunges and the inchworm. This was followed by 10 minutes of running or walking. Forty-minutes of high-intensity cardio-resistance training was then performed, including: pull-ups, kettlebell 2-arm swings, battle ropes, jump rope, jumping jacks, burpees, twisting, cross-punching, skating maneuvers, wide hips, body bar squats, medicine ball curl, resistance bands, full Turkish getups, soft medicine ball slams, dot drills, ladders, speed sack sprints, medicine ball chest pass, sandbag centipede drill, medicine ball lateral toss, and a parachute run. Notably, several of these tasks required coordinated social behavior (e.g., battle ropes, and medicine ball chest pass), engaging physical and social skills that are unique to this intervention modality. The final 10 minutes of the session were devoted to yoga-inspired flexibility training, static stretching, and a cool-down routine. Participants wore a heart rate monitor for each session, though they were not instructed to reach or maintain a particular heart rate during the training.

*Cognitive training.* Participants in the cognitive training session used a tablet computer to play *Mind Frontiers*, a suite of 7 Western-themed cognitive tasks with adaptive difficulty. Table 2 (in the main body of the paper) lists each task along with cognitive abilities targeted, including executive functions, visuospatial working memory, and analogical reasoning and the associated literature references. “Ante Up” required participants to match a configuration of a deck of cards with a set number of moves and is based on the Tower of London planning task. “Pen 'Em Up” required participants to sort items based on learned rules, drawing upon dual task-switching and executive function. “Supply Run” required participants to fill orders for items (within themed categories) and to engage working memory to remember the last item requested from each category. “The Irrigator” task required participants to use pipes randomly chosen from a set of configurations (straight, 90-degree elbow, t-shape, etc.) to connect a starting and ending point on a 2-dimensional grid and is a test of visuospatial reasoning. “Riding Shotgun” required participants to memorize target locations within a complex presentation of five 4 × 4 grids, arranged as a cross-hair, engaging visuospatial working memory. “Sentry Duty” is a dual n-back working memory task employing visual and auditory stimuli. “Trader Jack's” required participants to determine the value of an item compared to a target and engaged analogical reasoning. Performance in each mini-game yielded currency to spend on building a civilization in a meta-game component, a feature designed to promote motivation. A participant completed all 7 tasks for each training session.

For each *Mind Frontiers* task, each difficulty level is comprised of trials. Each Sentry Duty level had 21 items; but depending on the n-back level, there could be as few as 15 or as many as 20 trials per level. For some *Mind Frontiers* tasks, the number of trials per level was a difficulty manipulation. The number of trials in Irrigator is the number of wells in a level. For Supply Run the number of trials is the number of category bins. For Ante Up, each card movement could be considered a trial. All Mind Frontiers tasks start at Level 1, which also represents the minimum level. An accuracy of 100% (or nearly 100%) is required to trigger victory and advance one level higher for most *Mind Frontiers* tasks. Though a player can also stalemate (stay at the same level) or decrease a level, though Level 1 is the lowest. Supplementary Table 3 provides more details on the algorithm that determined whether a player would advance in difficulty level.

*Mindfulness meditation training.* The meditation sessions took place at a local yoga studio under the supervision of a licensed yoga instructor trained to administer the meditation protocol. At the start of the session, participants were provided 7 minutes to complete the Positive and Negative Affect Schedule (PANAS) survey, which is a self-report measure of affect over the past two days (i.e. assessing alertness, distress, irritability or determination). After completing the survey, participants were equipped with a heart-rate monitor and a wrist watch that communicated with the monitor. The next stage lasted 10 minutes and consisted of progressive relaxation and breathing until the participants were relaxed and this was followed by gentle stretching. Participants then engaged in seated silent meditation for 17 minutes, a 3-minute break allowing for deep breathing, light movement or stretching and then another meditation period lasting 15 minutes. A chime rang to begin and end meditation periods. After the second meditation period, participants lay flat on their backs for 5 minutes, which served as a recovery period. The session ended with each participant completing the PANAS again.

*Active control training.* Participants in the active control group engaged in computerized training tasks based on visual search and change detection training for 48 sessions over 16 weeks. The active control tasks were administered via tablet computers. The change detection task required the participant to identify the item that changed between two arrays of objects (i.e., cars, toys or street signs). Task difficulty increased with decreasing presentation time and/or increasing the number of objects in the array. The visual search task required participants to search for a target (i.e., F, P or a hand) among distractors. Difficulty was raised by increasing the number and/or heterogeneity of the distractors.

Supplementary Note 2.

**Adult Decision-Making Competence (A-DMC) battery.** We administered a well-validated battery of measures to investigate three facets of decision-making, employing the Adult Decision-Making Competence test: Value Assessment, Belief Assessment and Information Integration. The A-DMC was administered at pre- and post-intervention on a computer.

**Value Assessment**

*Resistance to framing.* Resistance to framing measures the extent to which a positive or negative framing of a problem can influence the evaluation of an outcome. Problems are either of a risky-choice or attribute type. At each cross-sectional administration, each participant saw pairs of questions that differed only in the positive or negative framing. For instance, suppose 1,200 endangered animals are threatened by a pesticide. The positive frame requires a choice between saving 600 animals for sure OR a 75% chance that 800 animals will be saved and a 25% chance that 0 animals will be saved. The negative frame is identical, except saving/saved becomes losing/lost.

*Resistance to sunken costs.* Resistance to sunken costs measures how well a participant ignores prior losses of time, money or other resources when deciding to continue investing in the sunk cost option (*52*). Responses are on a 6-point scale, where a ‘1’ response means the participant prefers the sunk-cost option whereas the ‘6’ response means the participant prefers the normatively correct option, which reflects the ability to ignore past loses and focus only on present consequences.

**Belief Assessment**

*Recognizing social norms.* Recognizing social norms measures how well a participant assesses social norms. There are two parts to each question. First, the respondent chooses from a binary response option for what they believe is socially acceptable. For instance, ‘Do you think it is sometimes okay to steal under certain circumstances?’. The second part asks, out of 100 age similar peers, how many would also find that behavior acceptable? The responses from all survey takers are aggregated from the first part of each question, providing an estimate of the percentage of respondents from the entire sample who endorsed the behavior.

*Over/Under confidence.* Over/under confidence measures the extent to which a participant recognizes the limits of their knowledge. A respondent first provides a true or false response to a question. Then the respondent is asked to quantify their confidence in their true or false response by rating that answer on a scale from 50% (just guessing) to 100% (absolutely sure).

*Consistency in risk perception.* Consistency in risk perception measures how well a participant adheres to probability rules. The participant scores the likelihood of an event happening to them on a scale between 0% (no chance) and 100% (certain). There are three dimensions manipulated to evaluate the respondent’s ability in assessing probability: time, complementariness and relational status (in set theoretic terms). For the time dimension, events are judged on their likelihood of happening in the next year or within five years. The one and five-year judgments by the participant are two separate items; but the pair is scored for the test as correct if the event happening in one year has a probability less than or equal to the event happening in five years. A complementary event is defined as one minus the probability of the event happening. For this dimension, there are two parts to each question, such as the probability of getting into an accident while driving and the probability of being accident free, and the probabilities assigned must sum to 1. The final dimension also contains two parts, a subset and superset event. If the superset event is the probability of death from any cause, then one possible subset event is the probability of death from a terrorist attack. Since the latter is a subset of the former, the latter must have a probability no larger than the former. If a respondent provides a response consistent with these set theoretic rules, then the item is scored as correct for this subtest.

**Information Integration**

*Applying decision rules.* Applying decision rules asks respondents to use different types of decision rules: elimination by aspects, satisficing, lexicographic, or equal weights. Each question has a set of consumer priorities and a set of product options under consideration by the decision-maker. Respondents select one answer from a set of multiple-choice options.

Supplementary Note 3.

**Tests of executive function and processing efficiency.** To assess whether the intervention training improved cognitive abilities, we used several test measures. The Set Shifting Task was used to test for gains in executive function. See Supplementary Figure 1 for more details. Processing efficiency was assessed using three measures. See Supplementary Figure 2 for more details. All of these tests were administered on a computer, except the Digit Symbol Substitution Task, which was paper and pencil.

Supplementary Note 4.

**Study Attrition.** The consort diagram summarizing study enrollment is presented in Supplementary Figure 3. The level of attrition (46%) was higher than studies that have only a single session or that administer a single intervention modality and reflects the significant time commitment (i.e., 60 hours) and task demands (i.e., up to three intervention modalities) required to complete the study. We performed a variety of analyses to investigate whether attrition introduced systematic bias in the study results. First, we examined whether the characteristics of participants that dropped out significantly differed from those that completed the study, investigating performance on pre-intervention measures of executive function, processing efficiency, and decision-making competence. In each case, we observed no evidence for statistically significant differences between groups. Second, we investigated whether the attrition rate between each of the intervention groups significantly differed. We found that the attrition rate was broadly equivalent across intervention groups (χ^2^=0.36, *p*=0.95), providing evidence that attrition did not differentially affect one intervention group over another. Thus, although the attrition rate (46%) constitutes a limitation of the present study, our analyses suggest that this factor did not introduce systematic bias in the study finding (i.e., with respect to participant characteristics and the level of attrition between groups).

Supplementary Figure 1. Test of executive function


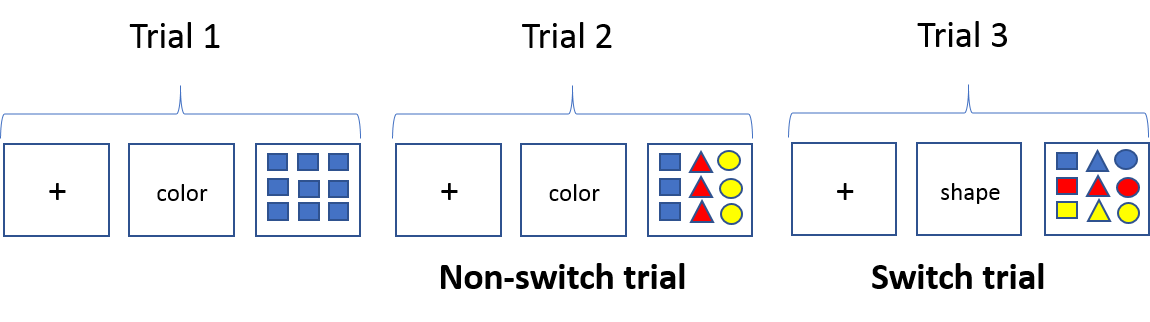


The Set Shifting Task (SST) was used to measure executive function. Each trial consisted of a fixation cross in the center of the screen, followed by a cue (color, shape or size) which denoted the feature of the visual stimuli array to pay attention to and, finally, the visual stimuli array. The figure below presents three trials. Trial 2 is a non-switch trial because the cue to pay attention to—color—remains the same between Trial 1 and Trial 2. But Trial 3 is a switch trial because the cue switches from color, in Trial 2, to shape, in Trial 3. The switch cost measure of executive function in the SST was determined by subtracting the number of correct responses on all non-switch trials from the number of correct responses on all switch trials. A switch trial occurs when the participant is cued to switch their focus from one attribute in the array of stimuli on a given trial to a different attribute on the next trial. In a non-switch trial, the attribute to focus on does not change between two trials. There were 2 sequential runs of 81 trials. In each run of 81 trials, 41 trials were switch and 40 were non-switch. For each trial, the fixation cross (ISI) was displayed, followed by the Cue (i.e. color, shape or size) for 1.2 seconds. The display was shown for 4 seconds, during which the participant needed to respond. The trial was 2.3 seconds in duration, but 3.5 seconds total from Cue to stimulus offset.

Supplementary Figure 2. Processing efficiency measures


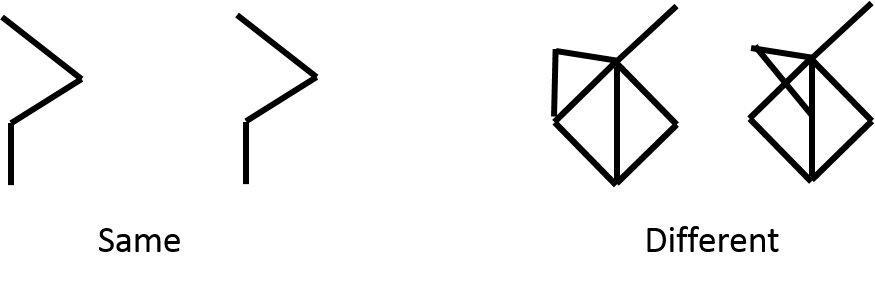


Three tests were administered to assess processing efficiency: Pattern Comparison, Letter Comparison, and the Digit Symbol Substitution Test. The above figure represents two trials from the Pattern Comparison task, where each trial consisted of two similar configurations of line segments. A decided if the configurations were the same or different and would put a ‘S’ if they were the same or a ‘D’ if they were different. (The words ‘Same’ and ‘Different’ included in the figure above were not included in actual test trials.) Letter Comparison was similar to Pattern Comparison. Participants determined whether two strings of letters were the same or different. Finally, the Digit Symbol Substitution test first provided a code table, which included a unique symbol for each digit between 0-9. The test was a lookup task where a participant was given a digit and had to provide the symbol corresponding to the digit.

Supplementary Figure 3. Consort diagram


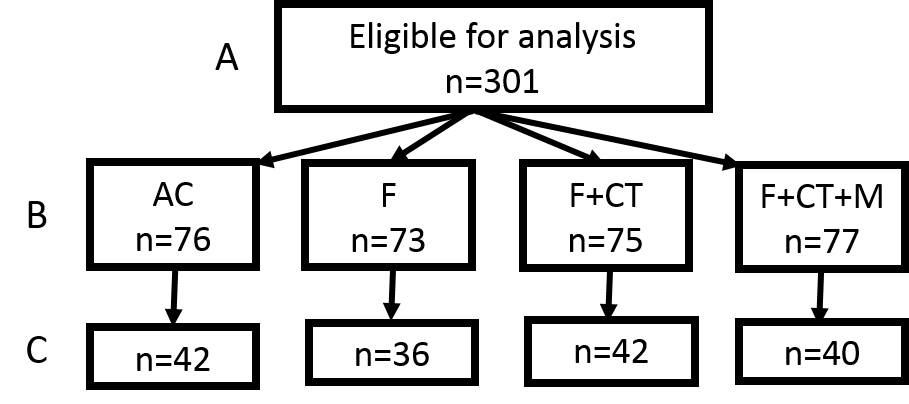


A. 301 participants completed the full battery of tests included in this study at pre-test. B. The 301 participants were randomly assigned to one of four intervention groups. ‘AC’ is Active Control; ‘F’ is Fitness; ‘F+CT’ is Fitness plus Cognitive Training; and ‘F+CT+M’ is Fitness plus Cognitive Training plus Meditation. C. 160 participants were enrolled at post-testing. Attrition did not differ across the four intervention groups (χ^2^=0.36, *p*=0.95).

Supplementary Figure 4. Cognitive training learning curves. For each of the 7 figures, the Average Difficulty (y-axis) represents the average game level achieved for a given session. Supplementary Table 3 provides the criteria to advance one level for each game.


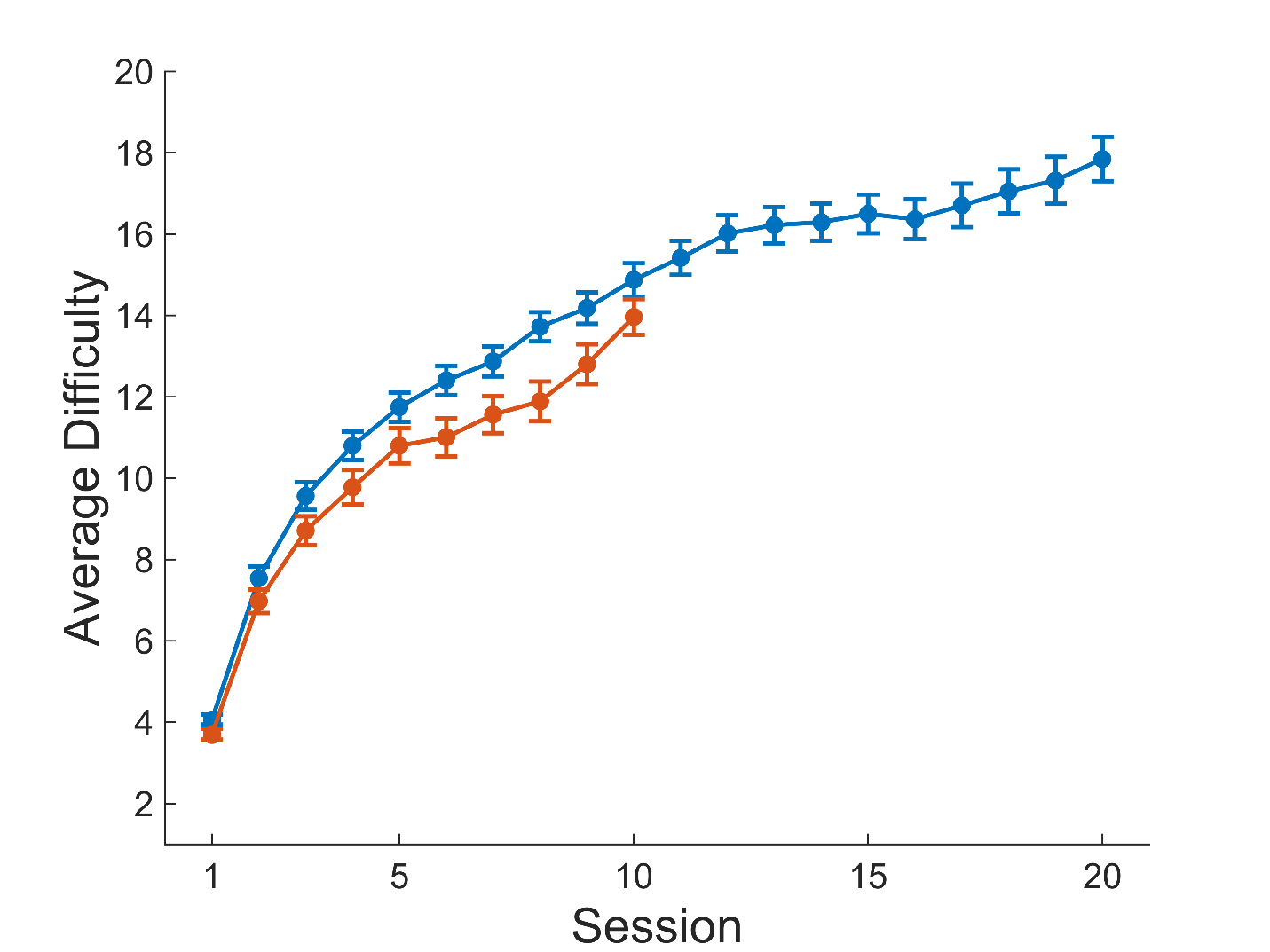


Session average by group for the Supply Run cognitive training task. The aerobic fitness plus cognitive training intervention completed 20 sessions (blue curve) and the aerobic fitness plus cognitive training plus meditation intervention completed 10 sessions (red curve). Error bars represent standard error of the mean.


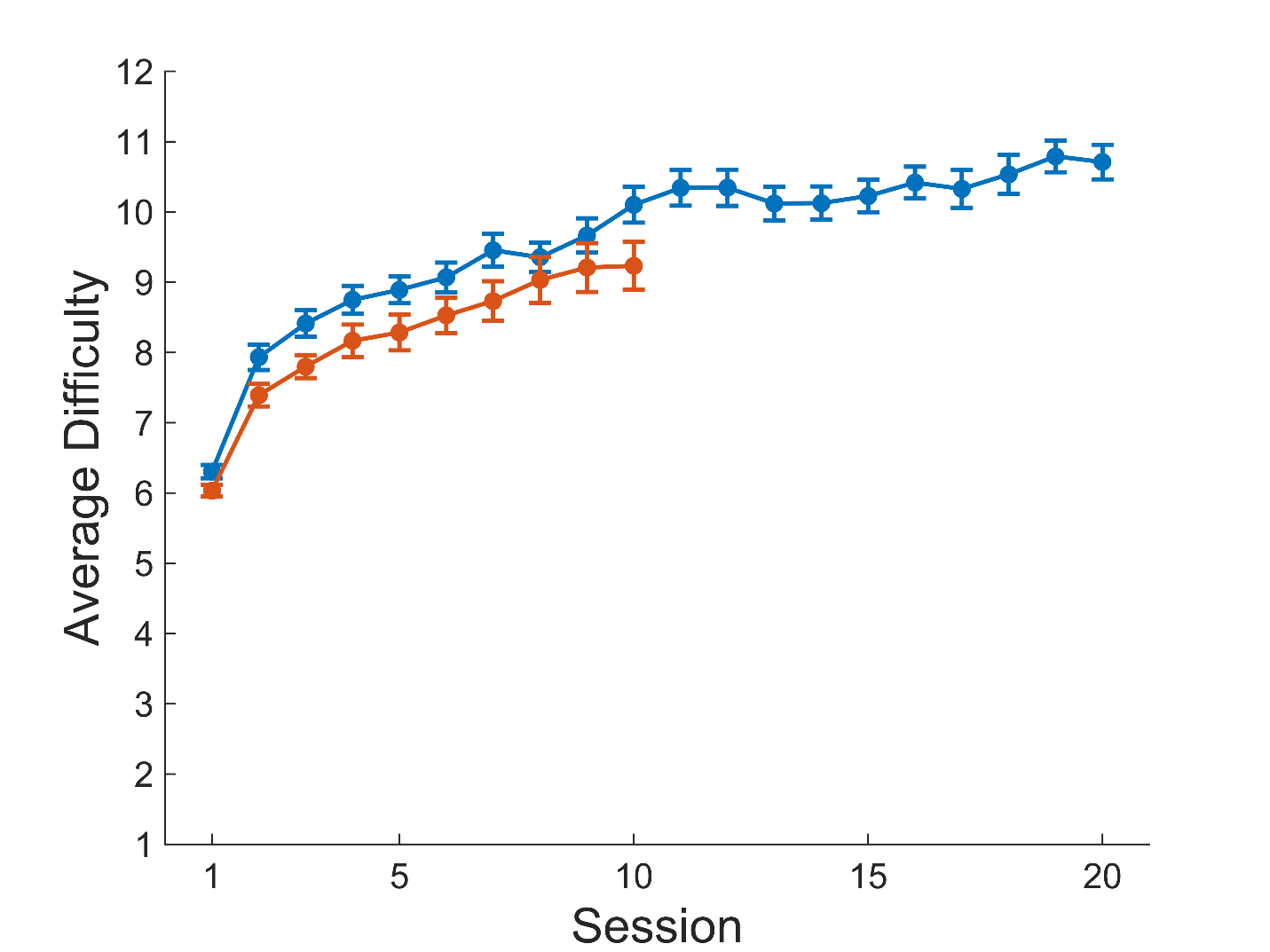


Session average by group for the Riding Shotgun cognitive training task. The aerobic fitness plus cognitive training intervention completed 20 sessions (blue curve) and the aerobic fitness plus cognitive training plus meditation intervention completed 10 sessions (red curve). Error bars represent standard error of the mean.


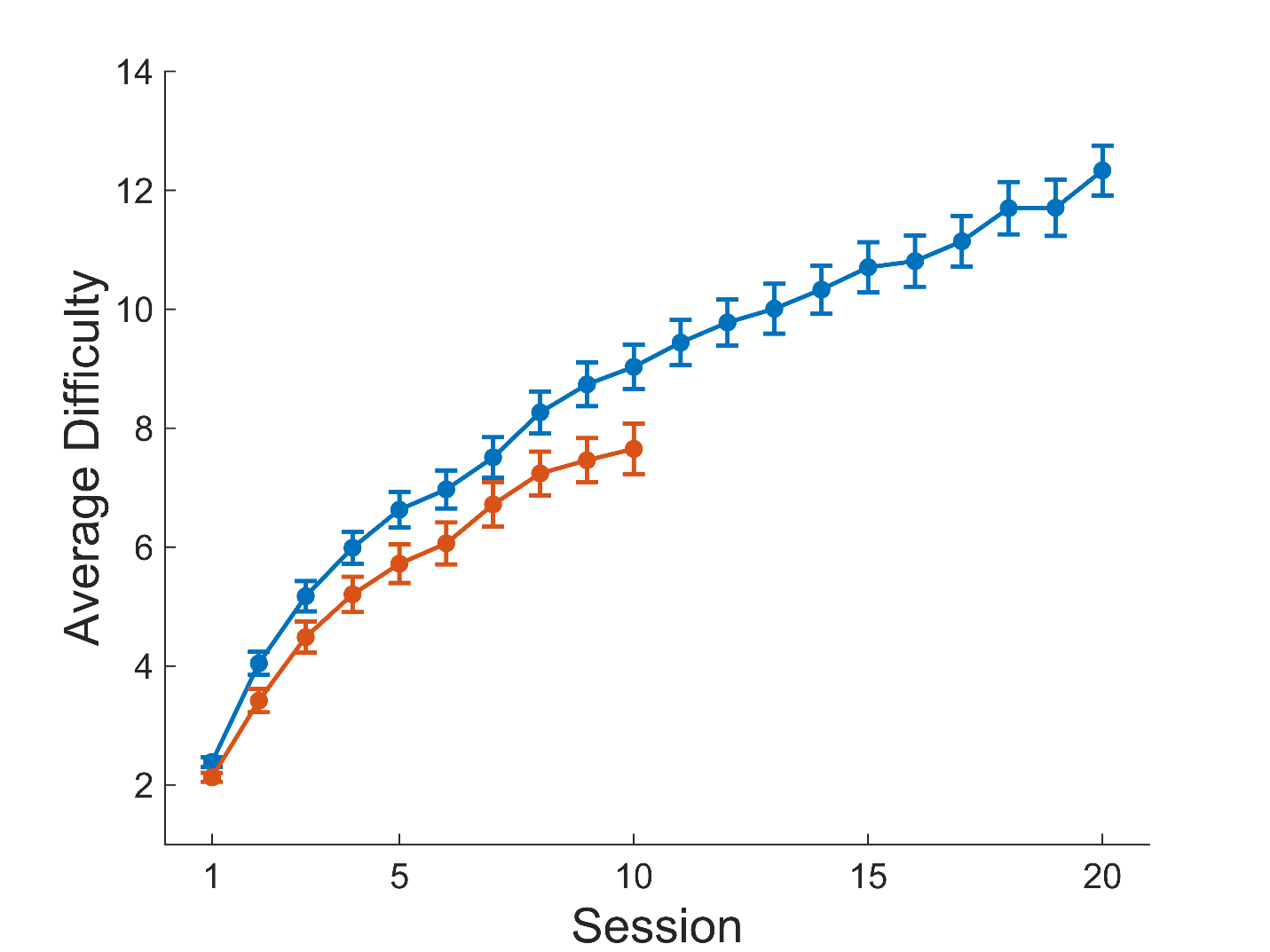


Session average by group for The Irrigator (or Pipe Mania), cognitive training task. The aerobic fitness plus cognitive training intervention completed 20 sessions (blue curve) and the aerobic fitness plus cognitive training plus meditation intervention completed 10 sessions (red curve). Error bars represent standard error of the mean.


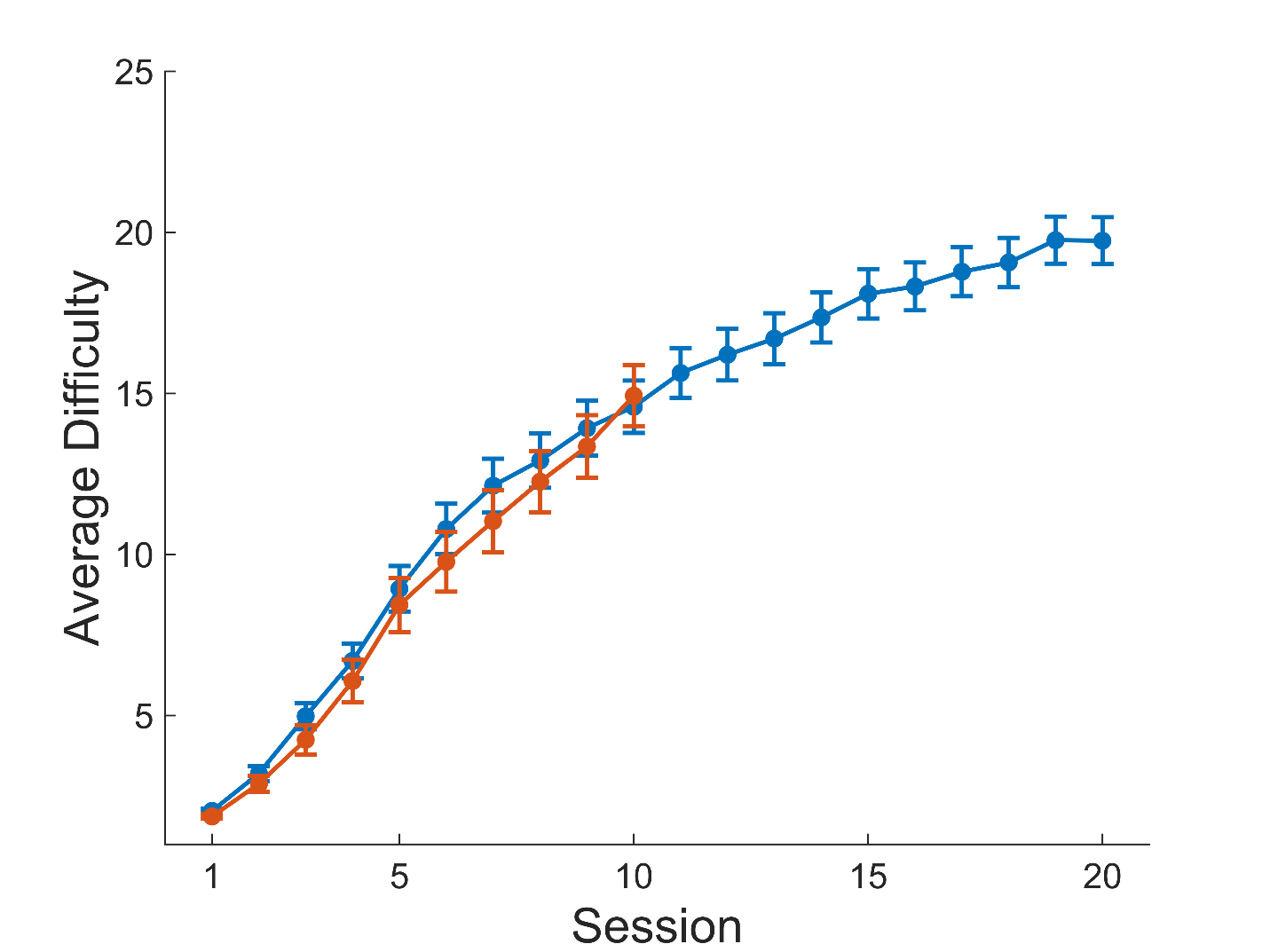


Session average by group for the Pen ‘Em Up cognitive training task. The aerobic fitness plus cognitive training intervention completed 20 sessions (blue curve) and the aerobic fitness plus cognitive training plus meditation intervention completed 10 sessions (red curve). Error bars represent standard error of the mean.


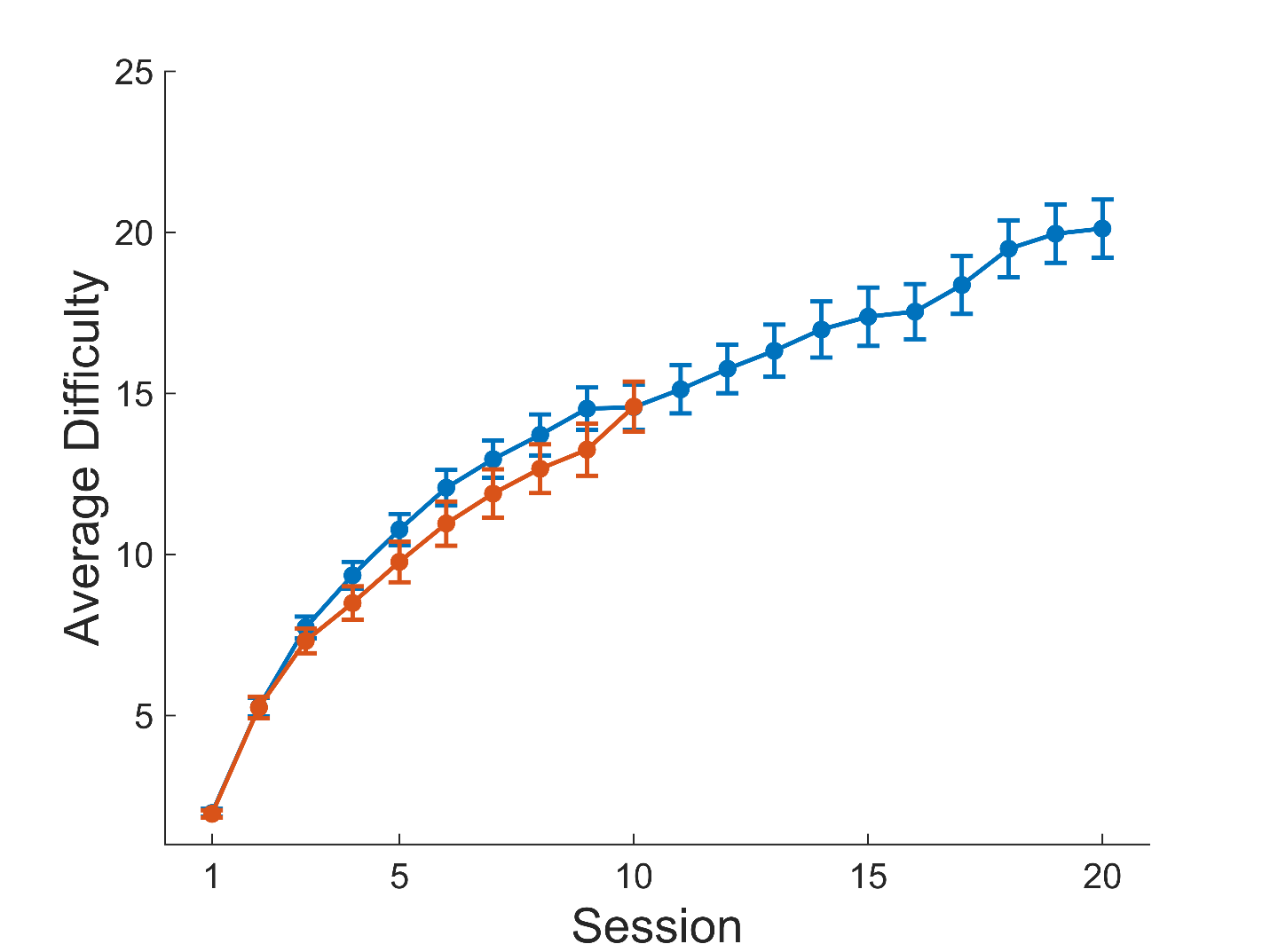


Session average by group for the Sentry Duty cognitive training task. The aerobic fitness plus cognitive training intervention completed 20 sessions (blue curve) and the aerobic fitness plus cognitive training plus meditation intervention completed 10 sessions (red curve). Error bars represent standard error of the mean.


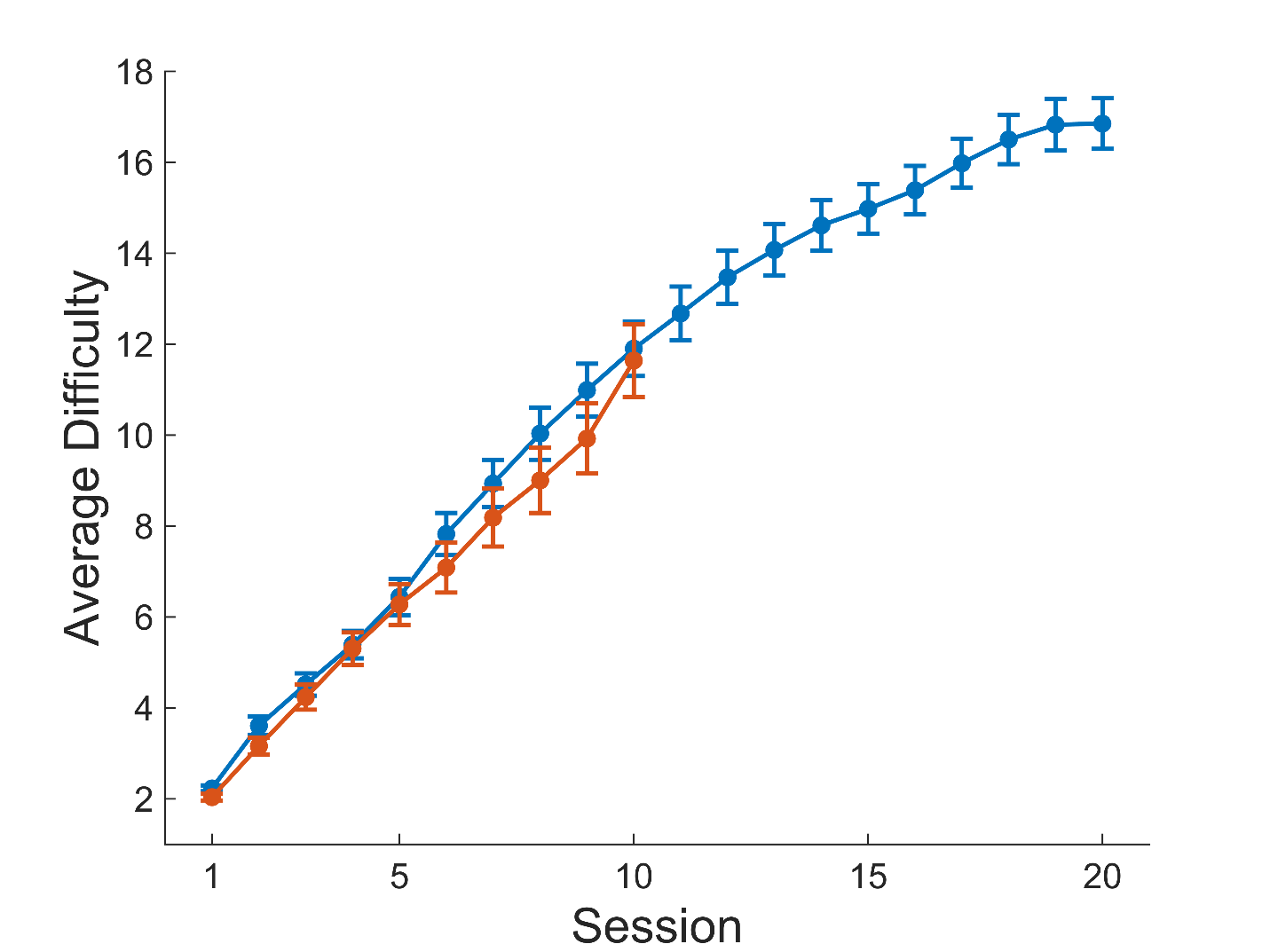


Session average by group for the Trader Jack’s (or Figure Weights) cognitive training task. The aerobic fitness plus cognitive training intervention completed 20 sessions (blue curve) and the aerobic fitness plus cognitive training plus meditation intervention completed 10 sessions (red curve). Error bars represent standard error of the mean.


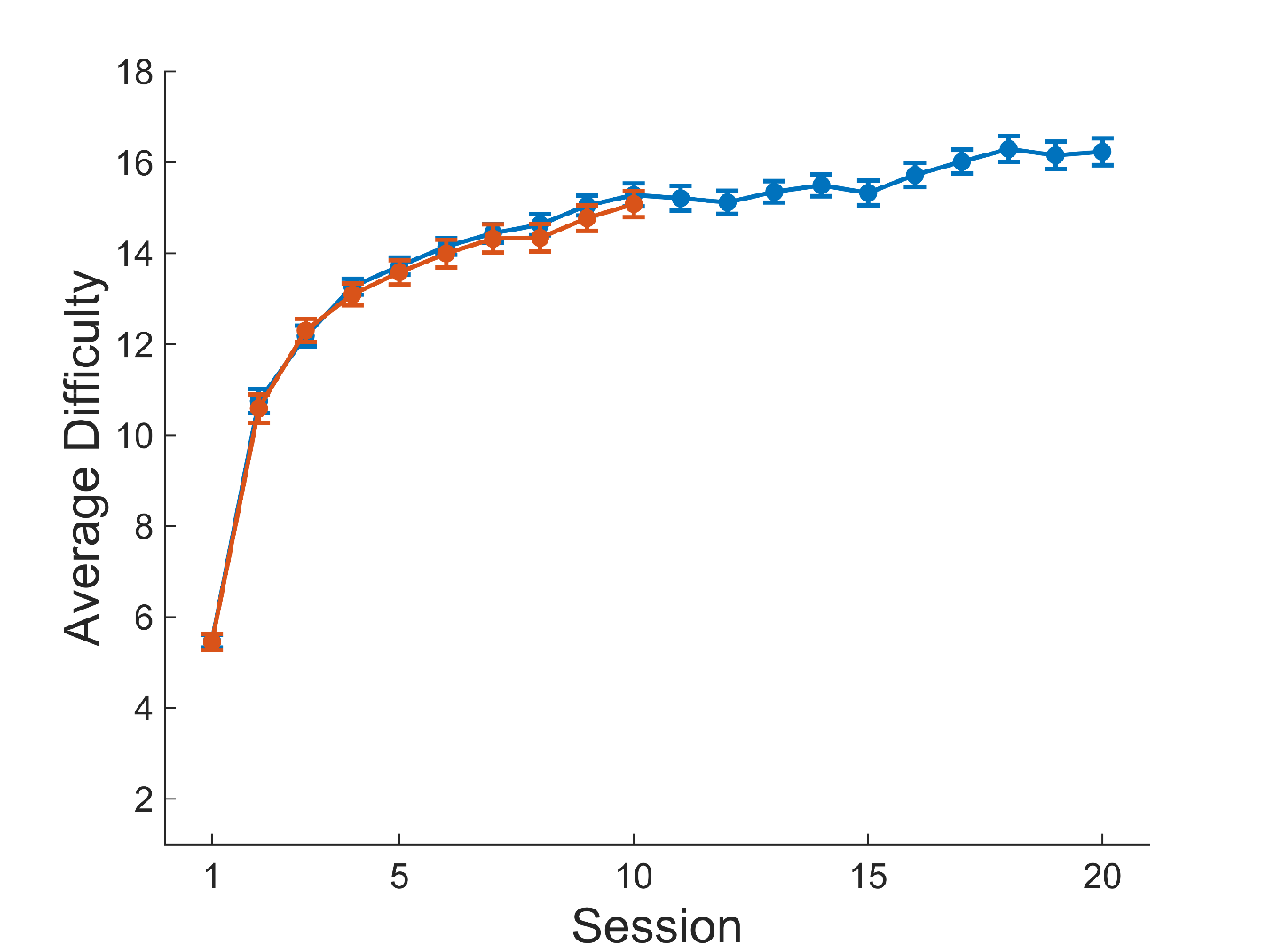


Session average by group for the Ante Up cognitive training task. The aerobic fitness plus cognitive training intervention completed 20 sessions (blue curve) and the aerobic fitness plus cognitive training plus meditation intervention completed 10 sessions (red curve). Error bars represent standard error of the mean.

Supplementary Figure 5. Active control learning curves


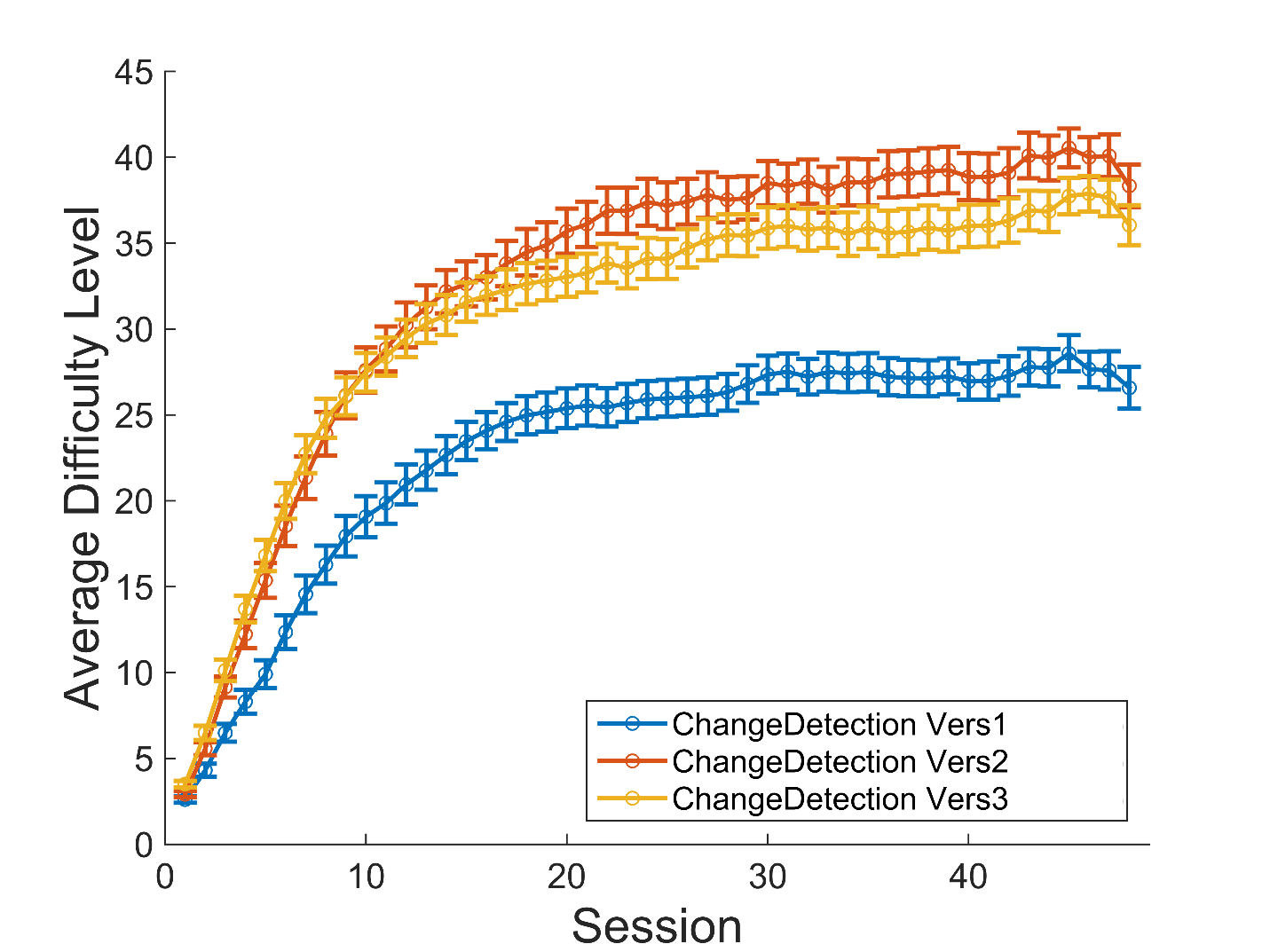


Session average for the three versions of the active control change detection task. Error bars represent standard error of the mean.


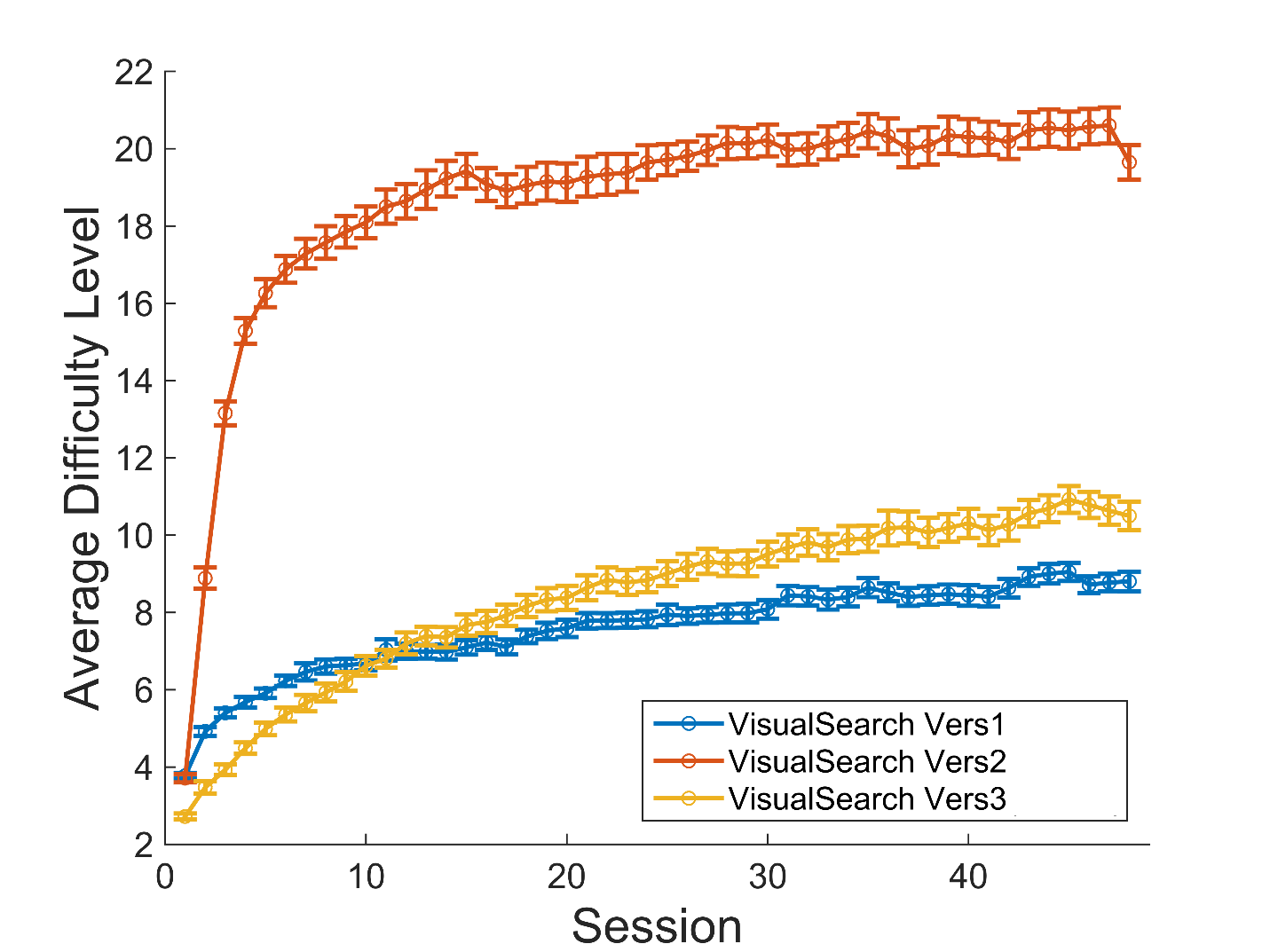


Session average for the three versions of the active control visual search task. Error bars represent standard error of the mean.

Supplementary Table 1.

Sequence of administration of each training modality for each intervention condition. ‘Session’ represents the training session number; ‘Fitness’ is the uni-modal high-intensity cardioresistance fitness training; ‘Fitness + Cognitive Training’ is the multi-modal high-intensity cardioresistance fitness and cognitive training group; ‘Fitness + Cognitive Training + Meditation’ is the multi-modal high-intensity cardioresistance fitness and cognitive training, coupled with mindfulness meditation training group; ‘Active Control’ is the active control training group. For the ‘Active Control’ column, ‘VS’ represents Visual Search and ‘CD’ represents Change Detection.

Supplementary Table 2. Regression coefficients and Cohen’s d effects sizes for each A-DMC subtest for each uni- and multi-modal intervention.

|  | F | F + C | F + C +M | AC |  |
| --- | --- | --- | --- | --- | --- |
| **Resistance to Framing** |  |  |  |  |  |
| Pretest mean (SD) | 4.30 (0.41) | 4.23 (0.40) | 4.13 (0.45) | 4.30 (0.39) |  |
| Posttest mean (SD) | 4.25 (0.38) | 4.30 (0.40) | 4.29 (0.33) | 4.29 (0.33) |  |
| Beta coefficients | -0.04 | 0.03 | 0.05 | -- |  |
| p-value | 0.60 | 0.70 | 0.52 | -- |  |
| Cohen’s d effect size | 0.01 | 0.01 | 0.02 | -- |  |
| **Resistance to Sunken Costs** | |  |  |  |  |
| Pretest mean (SD) | 4.20 (0.67) | 4.39 (0.68) | 4.28 (0.55) | 4.39 (0.81) |  |
| Posttest mean (SD) | 4.13 (0.78) | 4.46 (0.71) | 4.48 (0.60) | 4.17 (0.72) |  |
| Beta coefficients | 0.095 | 0.29 | 0.38 | -- |  |
| p-value | 0.45 | 0.017 | 0.002 | -- |  |
| Cohen’s d effect size | 0.04 | 0.42** | 0.62*** | -- |  |
| **Recognizing Social Norms** | |  |  |  |  |
| Pretest mean (SD) | 0.48 (0.21) | 0.47 (0.18) | 0.48 (0.25) | 0.48 (0.18) |  |
| Posttest mean (SD) | 0.57 (0.17) | 0.53 (0.20) | 0.52 (0.18) | 0.44 (0.20) |  |
| Beta coefficients | 0.13 | 0.09 | 0.08 | -- |  |
| p-value | 0.0035 | 0.042 | 0.068 | -- |  |
| Cohen’s d effect size | 0.59*** | 0.32** | 0.27* | -- |  |
| **Over Under Confidence** |  |  |  |  |  |
| Pretest mean (SD) | 0.92 (0.06) | 0.92 (0.07) | 0.92 (0.06) | 0.92 (0.05) |  |
| Posttest mean (SD) | 0.93 (0.07) | 0.91 (0.06) | 0.91 (0.06) | 0.92 (0.06) |  |
| Beta coefficients | 0.012 | 0.001 | -0.001 | -- |  |
| p-value | 0.32 | 0.96 | 0.49 | -- |  |
| Cohen’s d effect size | 0.08 | 0.00 | 0.03 | -- |  |
| **Consistency Risk Perception** | |  |  |  |  |
| Pretest mean (SD) | 0.77 (0.08) | 0.76 (0.09) | 0.73 (0.08) | 0.77 (0.10) |  |
| Posttest mean (SD) | 0.78 (0.09) | 0.77 (0.10) | 0.74 (0.11) | 0.75 (0.10) |  |
| Beta coefficients | 0.031 | 0.026 | 0.001 | -- |  |
| p-value | 0.16 | 0.22 | 0.93 | -- |  |
| Cohen’s d effect size | 0.17* | 0.12* | 0.00 | -- |  |
| **Applying Decision Rules** |  |  |  |  |  |
| Pretest mean (SD) | 0.89 (0.10) | 0.88 (0.09) | 0.87 (0.11) | 0.87 (0.10) |  |
| Posttest mean (SD) | 0.91 (0.09) | 0.89 (0.07) | 0.87 (0.10) | 0.85 (0.10) |  |
| Beta coefficients | 0.052 | 0.029 | 0.018 | -- |  |
| p-value | 0.012 | 0.14 | 0.37 | -- |  |
| Cohen’s d effect size | 0.47** | 0.18* | 0.06 | -- |  |
| **Composite z-score** | |  |  |  |  |
| Pretest mean (SD) | 0.17 (0.50) | 0.13 (0.48) | 0.04 (0.47) | 0.20 (0.48) |  |
| Posttest mean (SD) | 0.22 (0.48) | 0.19 (0.44) | 0.10 (0.31) | 0.00 (0.45) |  |
| Beta coefficients | 0.24 | 0.23 | 0.19 | -- |  |
| p-value | 0.0032 | 0.0032 | 0.015 | -- |  |
| Cohen’s d effect size | 0.59*** | 0.58*** | 0.43** | -- |  |

‘F’ denotes the high-intensity cardioresistance fitness training intervention; ‘F+C’ denotes the high-intensity cardioresistance fitness training plus cognitive training intervention; ‘F+C+M’ denotes the high-intensity cardioresistance fitness training plus cognitive training plus meditation intervention; and ‘AC’ denotes the active control. The active control was always the reference group, so all values presented here represent changes, relative to the active control. One asterisk denotes a small effect size (0.10 to 0.29), two asterisks denote a medium effect size (0.30 to 0.49) and three asterisks denote large effect sizes (>0.49). The Composite z-score was computed by averaging all 6 ADMC subtests and then z-scoring.

Supplementary Table 3. Cognitive training algorithm determining advancement in difficulty level for each *Mind Frontiers* training task.

| *Mind Frontiers* | Defeat  (Down 1 level) | Stalemate  (Same Level) | Victory  (Up one level) |
| --- | --- | --- | --- |
| Ante Up | <100% target,  <100% moves | 100% target,  <100% moves | 100% target,  100% moves |
| Figure Weights | 0% accuracy | 1-99% accuracy | 100% accuracy |
| Sentry Duty | <80% accuracy | 80-89% accuracy | >89% accuracy |
| Pen Em Up | <73% accuracy | 73-92% accuracy | >93% accuracy |
| Pipemania | <100% accuracy | --- | 100% accuracy |
| Riding Shotgun | <75% accuracy | 75-99% accuracy | 100% accuracy |
| Supply Run | <75% accuracy | 75-99% accuracy | 100% accuracy |

All Mind Frontiers tasks start at Level 1, which also represents the minimum level. An accuracy of 100% (or nearly 100%) was required to trigger victory and advance one level higher for most *Mind Frontiers* tasks. But there are some nuances to this general rule for some *Mind Frontiers* tasks, which are listed here. For Sentry Duty, Victory occurs by missing 2 or less items, Stalemate occurs with 3 or 4 wrong and Defeat occurs with 5 or more incorrect. Depending on the n-back (i.e. 1-back, 2-back, 3-back, 4-back, 5-back), the number of total items ranges between 15 and 20. So for 3-back, there would be 18 trials, and Victory occurs with at least 16/18 correct, which is 89%. For Pen 'Em Up, the length of the sequence increases by one item for each level, after Level 15. Victory occurs with no more than 1 trial incorrect, a Stalemate occurs with 2-4 incorrect and Defeat occurs with 5 or more wrong. So, for Levels 1-15, 93.3% is the cutoff for Victory; but the cutoff increases to 93.75% at Level 16, 94.1% at Level 17, 94.4% at Level 18, 94.7% at Level 19, 95% at level 20, etc. For Ante Up, a Stalemate is achieved by replicating the card stacks (and not the minimum number of moves). A player only get 3 extra moves for each Stalemate. If a player exceeds the number of extra moves, or cannot replicate the card stack, the result is Defeat. For Trader Jack's, Levels 1-19 have 3 trials per level, and levels 20-26 have 4 trials. The information in the table above is correct for Levels 1-19; but for Levels 20-26, 100% accuracy is needed for a Victory, 25-99% accuracy is needed to Stalemate and 25% accuracy results in Defeat.

Supplementary Table 4. Descriptive and inferential statistics for three facets of decision competence with aerobic fitness as the control group.

|  | F | F + C | F + C +M |
| --- | --- | --- | --- |
| **Value Assessment** |  |  |  |
| Pretest mean (SD) | 4.25 (0.38) | 4.31 (0.42) | 4.21 (0.33) |
| Posttest mean (SD) | 4.19 (0.51) | 4.38 (0.46) | 4.38 (0.32) |
| Beta coefficients | -- | 0.15 | 0.22 |
| p-value | -- | 0.09 | 0.01* |
| Cohen’s d effect size | -- | 0.32 | 0.55 |
| **Belief Assessment** |  |  |  |
| Pretest mean (SD) | 0.73 (0.085) | 0.72 (0.077) | 0.71 (0.092) |
| Posttest mean (SD) | 0.76 (0.061) | 0.74 (0.082) | 0.72 (0.062) |
| Beta coefficients | -- | -0.021 | -0.036 |
| p-value | -- | 0.20 | 0.029* |
| Cohen’s d effect size | -- | 0.20 | 0.45 |
| **Integration** |  |  |  |
| Pretest mean (SD) | 0.87 (0.103) | 0.88 (0.095) | 0.87 (0.108) |
| Posttest mean (SD) | 0.91 (0.085) | 0.89 (0.072) | 0.87 (0.099) |
| Beta coefficients | -- | -0.023 | -0.035 |
| p-value | -- | 0.25 | 0.08 |
| Cohen’s d effect size | -- | 0.18 | 0.33 |
| **Composite z-score** |  |  |  |
| Pretest mean (SD) | 0.17 (0.50) | 0.13 (0.48) | 0.04 (0.47) |
| Posttest mean (SD) | 0.22 (0.48) | 0.19 (0.44) | 0.10 (0.31) |
| Beta coefficients | -- | -0.008 | -0.053 |
| p-value | -- | 0.93 | 0.53 |
| Cohen’s d effect size | -- | 0.00 | 0.06 |

‘F’ denotes the high-intensity cardioresistance fitness training intervention; ‘F+C’ denotes the high-intensity cardioresistance fitness training plus cognitive training intervention; ‘F+C+M’ denotes the high-intensity cardioresistance fitness training plus cognitive training plus meditation intervention. F is the reference group in the ANCOVA model so all model betas, p-values and effect sizes represent changes, relative to the fitness group. Results with an * denote a significant p-value at the 0.05 threshold. This table, which uses the aerobic fitness intervention as the control, is the analogue to Table 4 in the manuscript, which uses the active control as the control in the ANCOVA model. The Composite z-score was computed by averaging all 6 ADMC subtests and then z-scoring.
